# A review of machine learning and deep learning algorithms for Parkinson's disease detection using handwriting and voice datasets

**DOI:** 10.1016/j.heliyon.2024.e25469

**Published:** 2024-02-05

**Authors:** Md.Ariful Islam, Md.Ziaul Hasan Majumder, Md.Alomgeer Hussein, Khondoker Murad Hossain, Md.Sohel Miah

**Affiliations:** a Department of Robotics and Mechatronics Engineering, University of Dhaka, Nilkhet Rd, Dhaka, 1000, Bangladesh; b Institute of Electronics, Bangladesh Atomic Energy Commission, Dhaka, 1207, Bangladesh; c Department of Electrical and Electronic Engineering, University of Dhaka, Dhaka, 1000, Bangladesh; d Moulvibazar Polytechnic Institute, Bangladesh

**Keywords:** Parkinson's disease (PD), Deep learning (DL), Machine learning (ML), Disease prediction, Diagnosis

## Abstract

Parkinson's Disease (PD) is a prevalent neurodegenerative disorder with significant clinical implications. Early and accurate diagnosis of PD is crucial for timely intervention and personalized treatment. In recent years, Machine Learning (ML) and Deep Learning (DL) techniques have emerged as promis-ing tools for improving PD diagnosis. This review paper presents a detailed analysis of the current state of ML and DL-based PD diagnosis, focusing on voice, handwriting, and wave spiral datasets. The study also evaluates the effectiveness of various ML and DL algorithms, including classifiers, on these datasets and highlights their potential in enhancing diagnostic accuracy and aiding clinical decision-making. Additionally, the paper explores the identifi-cation of biomarkers using these techniques, offering insights into improving the diagnostic process. The discussion encompasses different data formats and commonly employed ML and DL methods in PD diagnosis, providing a comprehensive overview of the field. This review serves as a roadmap for future research, guiding the development of ML and DL-based tools for PD detection. It is expected to benefit both the scientific community and medical practitioners by advancing our understanding of PD diagnosis and ultimately improving patient outcomes.

## 1 Introduction

**Fig. 1:**
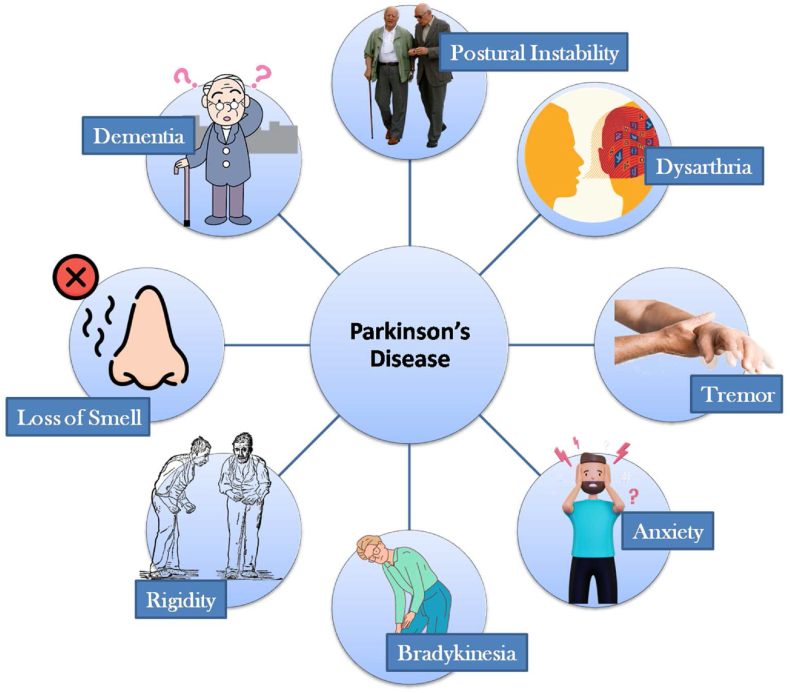
Parkinson's disease (PD) manifests itself in significant ways, both motor and non-motor.

Parkinson's disease (PD) is a chronic and progressive disorder charac-terized by the gradual loss of neurons in the substantia nigra, impacting the production of crucial neurotransmitters such as acetylcholine and sero-tonin [1]. These neurotransmitters play a vital role in controlling movement, and thus, PD predominantly affects motor function. The disease typically evolves through five stages. In stage 1, mild symptoms such as tremors and minor mobility issues emerge but do not significantly hinder daily function-ing. Stage 2 is marked by increased tremors and rigidity, making everyday tasks more challenging. At stage 3, balance and dexterity are compromised, leading to frequent accidents, yet individuals can often adapt to these difficul-ties. Stage 4 presents severe and debilitating symptoms, requiring assistance with daily activities. In the final stage, patients lose the ability to stand or walk and may experience delusions, often relying on wheelchairs for mobility. Beyond its direct impact, PD also poses a significant threat to the overall quality of life and increases the risk of developing other chronic diseases [2]. Diagnosing PD primarily relies on identifying both motor and non-motor symptoms. Fig. 1 illustrates the key motor and non-motor manifestations of PD. Distinguishing PD from other neurological disorders with similar eti-ology can be challenging, particularly as 75 % of PD cases are idiopathic. To enhance diagnostic accuracy and assist clinicians in making informed deci-sions, there is a growing need for computerized approaches rooted in ML and DL [3]. These techniques hold the potential to improve diagnostic perfor-mance in PD and related conditions.

### 1.1 Machine learning (ML) and deep learning (DL)

The subfields of artificial intelligence (AI), ML and DL are dedicated to developing algorithms and models capable of learning from data and making informed judgments or predictions [4]. The ML algorithms can be cate-gorized into three main types: supervised learning, unsupervised learning and reinforcement learning [5]. Supervised learning involves training an al-gorithm on a labeled dataset with predetermined correct answers for each input, enabling it to make accurate predictions for new, unseen inputs [6]. Unsupervised learning focuses on identifying patterns or structures in unla-beled data [6], while reinforcement learning entails learning from interactions with an environment to maximize a reward signal.

Deep learning involves creating artificial neural networks (ANNs) [7] that emulate the structure and functioning of the human brain. These neural networks, composed of interconnected layers of nodes or neurons, excel at recognizing intricate patterns and relationships in data. The DL has found applications in various domains, including natural language processing, au-tonomous driving, and image and audio recognition. A key advantage of ML and DL is their ability to make predictions or decisions without explicit rule-based programming, relying instead on data-driven trends and connec-tions [7]. This makes them particularly valuable in industries like healthcare, where complex data can be challenging for humans to analyze. Successful application of these methods requires extensive data and computational re-sources for training and optimization.

The emerging field of artificial general intelligence aims to develop intelli-gent computers capable of performing diverse cognitive tasks akin to humans. Yang et al. (2015) [8] proposed a novel spike-based meta-learning approach designed to be robust to noisy and non-stationary data encountered in prac-tical applications. This approach learned a resilient meta-model capable of adapting to shifting data distributions over time using a constrained minimal error entropy criterion. Yang et al. (2022) [9] developed a unique spike-based learning algorithm that utilized heterogeneous ensembles of neural networks for few-shot online learning tasks, demonstrating exceptional accuracy, ro-bustness, and low power consumption compared to state-of-the-art DL mod-els [10]. enhanced the accuracy and resilience of spike-based learning al-gorithms by integrating context-dependent learning and fault-tolerant spike routing into a neuromorphic learning framework, achieving high accuracy with minimal power usage in various classification tasks.

This work focuses on the diagnosis of PD, a prevalent neurodegenerative condition impacting both motor and non-motor aspects [11]. It addresses two key challenges in PD diagnosis: the subjectivity of traditional diagnostic methods and the identification of early non-motor symptoms [12]. To ad-dress these challenges, the study explores the application of ML algorithms for classifying PD patients and healthy controls or patients with similar clini-cal presentations. This work provides a comprehensive review of the existing literature on ML and DL algorithms for PD diagnosis and differential diag-nosis.

### 1.2 Rationale

In recent years, there has been a noticeable surge in the number of articles focusing on the diagnosis of PD through the utilization of DL approaches, re-flecting a growing interest in this area as evidenced by the increasing number of published studies [13,14].

A systematic review conducted by Heidari et al. (2022) [15] employed Cochrane's seven-step methodology. Beginning with the formulation of re-search questions and establishment of inclusion or exclusion criteria, the re-searchers conducted comprehensive searches using pertinent keywords in var-ious databases. Subsequently, articles were selected, and relevant information was collected and aggregated. Following rigorous inclusion and exclusion cri-teria, 82 articles were selected from an initial pool of 10,980. The findings from this review highlight the prevalence of ML algorithms such as Random Forest (RF), Support Vector Machine (SVM), and Logistic Regression (10.13039/501100009319LR), which have demonstrated effectiveness in the diagnosis of PD.

ML algorithms have played a pivotal role in enhancing the detection and evaluation of PD, particularly in distinguishing individuals with PD from those with similar clinical manifestations [3,16]. Mei et al. (2021) [17] con-ducted a thorough assessment by evaluating articles published until February 14, 2020, using resources such as PubMed and IEEE Xplore. This compre-hensive review covered various aspects, including research objectives, data sources, data types, ML methodologies, and research findings. The study underscores the potential of ML, DL, and novel biomarkers in advancing the diagnosis of PD.

The study conducted by Moro et al. (2021) [18] delves into the predom-inant characteristics and methods used in PD diagnosis. It focuses on ML algorithms employing speech and voice analysis to rapidly identify and assess PD. The paper elaborates on the predictive capabilities of these models, high-lights published discoveries, and discusses common technical challenges that can impact results. The objective is to provide a comprehensive overview of these techniques, their advantages, limitations, and to identify promising directions for further research. Notably, the study emphasizes the signifi-cance of fricatives and apparent speech and voice features in computerized PD detection and severity evaluation. Given the absence of a clinically es-tablished standard method, the authors stress the need for further research, including the creation of larger data corpora and the identification of reliable biomarkers.

Classification in PD detection has been instrumental in saving time and improving diagnostic accuracy. Pahuja et al. (2018) [19] explored various classifiers aimed at enhancing outcomes in PD detection. However, the chal-lenge lies in determining the most effective classifier for this purpose. Com-paring different classifiers on local datasets can be complex. In their research, they conducted a comparison of Multi-Layer Perceptrons (MLP), SVM, and K-Nearest Neighbors (KNN) using a standardized voice dataset to ascertain which classifier offers the highest accuracy and efficiency for PD classification [5,20].

AI systems are gaining significant traction in the realm of medical diag-nostics due to their capacity to handle vast amounts of data and generate robust statistical predictions. Saravanan et al. (2022) [21] conducted a sys-tematic examination of the impact of ML and DL-based AI strategies on the diagnosis of PD. Additionally, this research delves into the current state of data-driven AI applications in PD diagnosis.

## 2 Objectives

Table 1 provides a comparative overview of the previous review works and the proposed review study, highlighting their coverage periods, the num-ber of studies and datasets analyzed, the presence of voice and handwriting datasets, and the number of machine learning methods considered.

**Table 1:** Comparison between previous review works and our proposed review study.

Authors	Coverage	Number of studies	Number of Dataset	Voice dataset?	Hand writing dataset?	#ML meth- ods
**Nader** **Salari 2022**	2012–2020	82	7	Yes	No	Not Given
**Jie Mei** **2021**	2009–2020	209	16	Yes	No	448
**Laureano** **Moro- Velazquez,** **2021**	1960–2020	192	Not Given	Yes	No	Not Given
**Gunjan** **Pahuja 2018**	Upto 2017	Not Given	1	Yes	No	3
**S. Sara-vanan 2021**	2009–2020		2	Yes	No	18
**Our study**	2000–2022	60	20	Yes	Yes	70

**Fig. 2:**
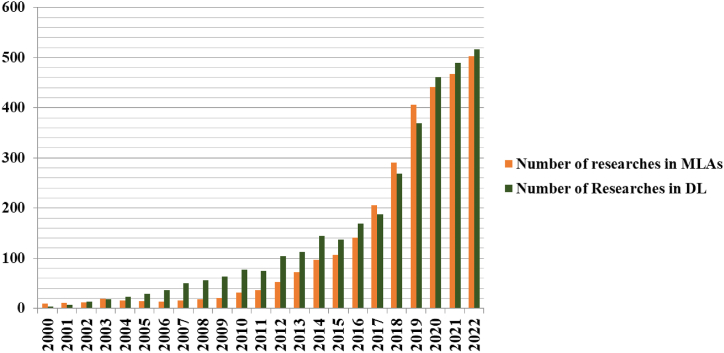
Techniques of machine learning and deep learning applied toward the diagnosis of Parkinson's disease.

The progression of ML and DL techniques in PD detection is depicted in Fig. 2, which covers the period from 2000 to March 2023 and features various studies published during that time span.

In our comprehensive literature survey, we systematically reviewed stud-ies published up until March 2023, utilizing databases such as IEEE Xplore and PubMed. Our examination encompassed the objectives of these studies, the data sources and types employed, as well as the ML and DL method-ologies utilized, along with their associated findings. One noticeable trend in recent years has been the increasing number of studies dedicated to the diagnosis of PD through DL methods. This surge in publications reflects the growing interest and exploration in this area. Our study not only eval-uated the existing research landscape but also introduced novel research di-rections for further exploration. To enhance the reliability and precision of PD diagnosis, we implemented a systematic evaluation framework. Within this framework, we compared the performance of various classifiers using the same dataset and features. This enabled us to identify the PD classifier with the highest accuracy, sensitivity, and specificity. Additionally, we employed ensemble techniques by combining the predictions of multiple classifiers to further enhance performance.

In our systematic review, we cast a wide net by accessing scientific databases such as PubMed, ERIC, JSTOR, IEEE Xplore, and Google Scholar. We examined a total of 60 studies published up to March 2023. Our review en-compassed an analysis of the studies’ objectives, data sources, data types, as well as the ML and DL methodologies employed. The collective find-ings from these studies strongly suggest that ML and DL-based approaches, alongside emerging biomarkers, hold immense potential in improving clinical decision-making processes. This, in turn, could lead to more comprehensive and precise diagnoses of PD.

## 3 Methodology

**Fig. 3:**
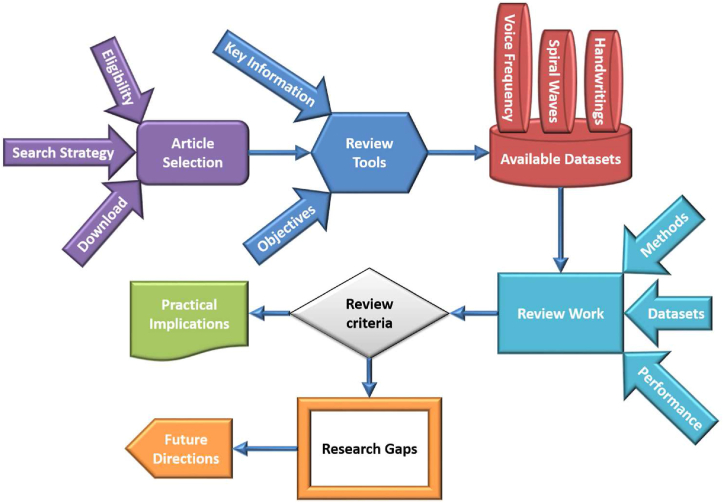
The process of review.

Fig. 3 shows the working flow diagram of this review paper. As can be seen in Fig. 3, the manner of the review process for this article is comprised of various different parts.

### 3.1 Search strategy

#### 3.1.1 Information sources

**Fig. 4:**
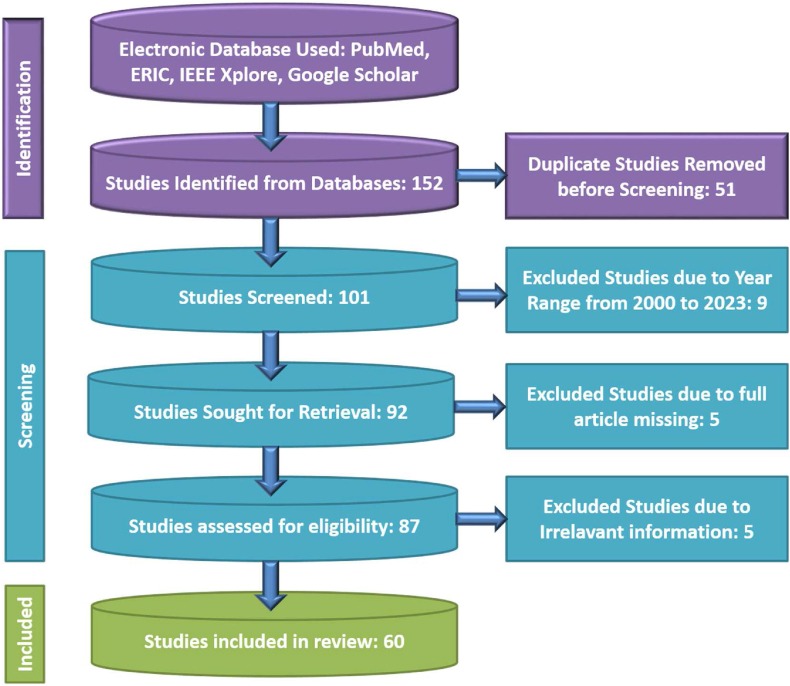
PRISMA flow diagram of the review work.

We conducted an extensive search for relevant literature using multiple reputable sources, including PubMed, ERIC, JSTOR, IEEE Xplore, and Google Scholar [22]. To identify pertinent journal articles, we employed spe-cific search keywords such as ”PD detection using ML,” ”PD detection using deep learning,” and ”PD detection.” All search results were taken into con-sideration. Our approach involved a meticulous and comprehensive review of each retrieved study. Following the guidelines outlined in the Preferred Reporting Items for Systematic Reviews and Meta-Analyses (PRISMA), we systematically screened and examined each study. This process enabled us to extract relevant data and information from the selected articles, contributing to the comprehensiveness of our review. For a visual representation of our review process, please refer to Fig. 4.

#### 3.1.2 Criteria for eligibility and ineligibility

For the purpose of this literature evaluation, we initially retrieved a total of 152 research publications from the five websites, based on the predefined criteria. These papers spanned publication years from January 2000 to March 2023 and were considered suitable for our review and discussion. A research team consisting of four researchers rigorously analyzed these 152 diverse re-search papers. To refine our selection and focus on the most relevant studies, we applied several criteria. Specifically, we sought out studies that employed ML and DL methods for PD detection. Given the nature of ML or DL, these approaches inherently involve automation. Furthermore, ML could be categorized into supervised, unsupervised, or reinforcement-based learning, while DL could be further categorized based on the data type, including structured data, images, or sequences. To further narrow down the selec-tion, we specifically looked for studies that used both handwriting images and voice datasets while applying ML and DL techniques. The criteria for inclusion encompassed the following.

• Studies employing supervised, unsupervised, or reinforcement-based ML for PD detection.
• Studies utilizing structured data, images, or sequences-based DL for PD detection.
• Research incorporating computer vision and wearable tools combined with ML or DL-based digital techniques for PD detection.

A predominant majority of researchers in the field have explored deep learning algorithms such as Convolutional Neural Networks (CNN), Recur-rent Neural Networks (RNN), Deep Neural Networks (DNN), Long Short- Term Memory (LSTM), and others, as well as supervised machine learning algorithms including ANN, LR, SVM, Decision Trees (DT), KNN, Fuzzy Networks, Feed forward Back propagation ANNs (FBANN), Naive Bayes (NB), RF, XGBoost, AdaBoost, Radial Basis Function (RBF), and ResNet, as documented in Table 5 and Table 6. A smaller percentage of researchers explored unsupervised or semi-supervised ML and DL algorithms like Prob-abilistic Neural Networks (PNN), Bidirectional Long Short-Term Memory (BLSTM), Graph Laplacian Regularized Autoencoders (GLRA), Deep Be- lief Networks (DBN), Improved Grey Wolf Optimizer (IGWO), Complex- Valued Multi-Layer Topology Adaptive Neural Network (CMTNN), Extreme.

**Table 2:** Performance metrics for evaluating the model.

Performance metrics	Definition	Number of Studies conducted
**Accuracy**	$\frac{TP+TN}{TP+TN+FP+FN}$	174
**Precision**	$\frac{TP}{TP+FP}$	31
**F1-score**	2 $\times \frac{precision\times recall}{precision+recall}$	25
**Recall**	$\frac{TN}{TN+FN}$	110

**Table 3:** Available datasets to conduct the review work.

Dataset	UCI Dataset	PPMI	HandPD	New HandPD	mPower Database	PaHaw	PDMultiMC
**Number of Instances**	197	600	736	264	898	75	32
**PD patients**	–	400	74	31	–	37	16
**Healthy controls**	–	200	18	35	–	38	16
**Number of Attributes**	23	159	16	–	–	8	16
**Time of release**	September 2008	December 2011	2016	2016	2016	2016	2018
**Number of papers cited**	1130	1345	204	204	337	290	32
**Data Set Characteristics**	Multivariate	Multivariate	Multivariate	Multivariate	Multivariate	Multivariate	Multivariate
**Data format**	Voice	Numerical and Categorical	Images	Images	Apps	Handwritings	Demographics
**Associated Tasks**	Classification	Classification	Classification	Classification	Classification	Classification	Classification
**Missing Values?**	N/A	N/A	N/A	N/A	N/A	N/A	N/A

**Table 4:** Comparison in features analysis (handwriting and voice recognition) from other researches.

Comparison in features analysis (handwriting and voice recognition) from other researches	
**Features of Voice Dataset**	**Features of Handwritten Dataset**
Fundamental frequency (Fo), High-frequency component (Fhi), Low-frequency component (Flo), Jitter percentage (%), Absolute jitter, RAP, PPQ, DDP, Local shimmer, Shimmer in decibels (dB), APQ3, APQ5, APQ, DDA, NHR, HNR, RPDE, DFA, Spread measure 1, Spread measure 2, D2, PPE [30,36] [37,38].	Stroke speed (mm/s), Speed (mm/s), Velocity (mm/s), Acceleration (mm/s^2^), Jerk rate (mm/s³), Horizontal ve-locity/acceleration/jerk, Vertical velocity/acceleration/jerk, NCV, NCA, Relative NCV, On-surface time [34,39].
The analysis encompasses a total of 44 acoustic attributes, systematically classified into five distinct types. These cat- egories include Pitch local perturbation measures, comprising jitter relative (expressed as a percentage), absolute jitter, jit- ter RAP, and jitter PPQ. Additionally, Amplitude local per- turbation measures involve shimmer local, shimmer in deci- bels (dB), APQ3, APQ5, and APQ11. Noise features, specif- ically HNR and GNE, constitute another category. Spectral envelope measures are represented by MFCCs (Mel-frequency cepstral coefficients), and Nonlinear attributes are captured through RPDE, DFA, and PPE. This structured classifica- tion provides a comprehensive framework for analyzing various acoustic characteristics [40,41].	The analysis involves several key parameters, including writing pressure, grip pressure, and ink refill pressure. Additionally, it encompasses metrics related to the radius of the pen tip, such as the root mean square (RMS) of the difference between the horizontal and vertical (HT and ET) radii, the maximum and minimum differences between HT and ET radii, the standard deviation (SD) between HT and ET radii, and the mean re- sultant time (MRT). Furthermore, the evaluation incorporates dynamic aspects, such as the number of times the difference between HT and ET radii changes sign, indicating shifts in the writing dynamics. This comprehensive set of metrics provides a detailed characterization of various aspects of the writing process, offering valuable insights into pressure variations, pen grip, and pen movement dynamics [25,42,43].
Professional microphone Channel-Yaffe (YA) feature, IS4- par-alaling compact, IS6-speaker trait, EM1 embose, Smartphone channel- KTU features, MP-MPEG7 descriptors, EM1-emboss [44].	X, Y, Pressure, Tilt-x, Tilt-y, Button status, Displacement, Velocity, Jerk, Acceleration, Horizontal/vertical displacement, velocity and acceleration, First derivative of pressure [45].
MFCC, WT, and TQWT [46].	X, Y, Z, pressure, altitude, azimuth, and time Stamp [47].
The dataset includes several demographic and technical pa-rameters. These encompass the mean age, gender distribution (M/F), the number of sensors per foot, the sampling rate (100 samples per second), the number of variations in fundamental frequency (Hz), and measures of amplitude variation in deci- bels (dB). This comprehensive set of information provides a diverse range of factors, incorporating both individual charac- teristics and technical specifications, which can be crucial for a thorough analysis or classification task [48].	The dataset encompasses various distance metrics, including Euclidean distance, relative distance, circular distance, and Manhattan distance. Additionally, it includes pixel similar- ity measurements, providing insights into spatial relationships. The parameters design speed and design time further con- tribute to the dataset, offering information about the tempo- ral and performance aspects of the analyzed entities. This collection of metrics presents a well-rounded set of measures, combining spatial and temporal characteristics, which can be valuable for diverse analytical purposes [49].
The dataset comprises various acoustic features related to voice analysis. These include MDVP: Fo (fundamental fre- quency in Hz), MDVP: Fhi (highest frequency in Hz), MDVP: Jitter (percentage), MDVP: PPQ (pitch period perturbation quotient), MDVP: Jitter (absolute), Jitter: DDP (jitter cycle- to-cycle difference), MDVP: RAP (jitter relative average per- turbation), MDVP: Shimmer, MDVP: Shimmer (in decibels), MDVP: APQ (amplitude perturbation quotient), Shimmer: APQ3, Shimmer: APQ5, Shimmer: DDA (discrete cosine transform amplitude), RDPE (recurrence period density en- tropy), HNR (harmonics-to-noise ratio), and NHR (noise-to- harmonics ratio). This diverse set of parameters captures dif- ferent aspects of voice modulation, providing a comprehensive basis for voice quality assessment [38,50].	x - and y -coordination, pen position, corresponding time coor-dinates, pen inclination, i.e. azimuth and altitude, and pres- sure exerted over the writing surface were recorded, Button status, in-air movement, and on-surface movement, Velocity, Acceleration [25,45,51].
Binary (256) and ternary pattern (512) of the voice signal from three diagnostic diseases (frontal resection, cordectomy, and spastic dysphonia) [52]	X-Y-Z coordinates, pressure, altitude, azimuth, time stamp, and grip angle [32,47].
Frequency parameters: Jitter (local, absolute, rap, ppq5, DDP); Amplitude parameter: Shimmer (local, dB, apq3, apq5, apq11, DDA) Voicing parameter: (frames, number and degree of voice breaks); Pitch parameters: (Mean, median, SD, Min- imum, and Maximum); Harmonicity parameters: (Autocorre- lation, NHR, HNR) [53]	The dataset encompasses a range of features related to en-tropy, energy, and empirical mode decomposition. In ad- dition, it includes parameters such as NCV (nerve conduc- tion velocity), NCA (nerve conduction amplitude), relative NCV/NCA, writing duration, length, stroke speed, veloc- ity, acceleration, jerk, horizontal velocity/acceleration/jerk, vertical velocity/acceleration/jerk, stroke height/width, wing phase, stance phase, and stride time. Furthermore, statistical measures like skewness, coefficient of variation, kurtosis, and correlation contribute to the comprehensive characterization of the dataset. This diverse set of features provides a holistic perspective, covering aspects of signal processing, motion dy- namics, and statistical properties for a thorough analysis [54].
GA and SVM are utilized to extract features such as Formant frequencies, Discrete wavelet transform, time-domain energy and ZCR, wavelet Shannon entropy, and MFCC from the voice dataset. (Zayrit Soumaya-2020)	RF1 to RF8, LF1 to LF8, RF and LF [55].
Pitch regional perturbation evaluation. Notional jitter, unvar-nished jitter, RAP, pitch perturbation section; Harmonic to noise rate measure; HNR, NHR, MFCC, PSE, RSIE [56]	Euclidean distance, relative distance, circular distance, Man-hattan distance, pixel similarity, design speed, and design time [49].
Continuation of … Comparison in features analysis (han	dwriting and voice recognition) from other researches
**Features of Voice Dataset**	**Features of Handwritten Dataset**
Pitch local perturbation, amplitude perturbation, harmonic-to-noise ratio, MFCC measures from order 0–12, Entropy of Recurrence period, DFA, PPE, Ratio of glottal-to-noise exci-tation [57].	Cartesian and XY features, Pen tip pressure feature, Azimuth and altitude feature, RMS value, Zero Crossing (ZC) index, Pattern specific features [58].
The dataset includes a variety of features related to speech analysis, encompassing speech frequency variations, sound ve- locity, pressure metrics, wavelength variations, and pitch varia- tions across multiple pronunciations, including letters, words, sentences, and numbers. These parameters collectively offer a detailed insight into the acoustic characteristics of speech, covering aspects of frequency modulation, sound propagation, pressure dynamics, and pitch variations across different lin- guistic units. This dataset is designed to provide a comprehen- sive foundation for studying the nuances of speech production and articulation in various contexts [59].	The dataset includes temporal parameters related to motion, such as in-air time and on-surface time, which represent the duration of a specific activity both in the air and on a surface. Additionally, it involves normalized counterparts, namely nor- malized in-air time and normalized on-surface time, providing a scaled perspective. The in-air/on-surface ratio further con- tributes by quantifying the relationship between time spent in the air and on the surface. This set of parameters offers a nuanced understanding of temporal dynamics, particularly during activities involving both airborne and surface interac-tions [34].

**Table 5:** Parkinson disease detection based on voice dataset.

	Parkinson disease detection based on	voice dataset	
**Dataset**	**Methodology**	**Accuracy**	**References**
The EEG signals of 20 PD patients	Classifier: 13 layers of CNN	88.25 %	[81]
Time Series Datasets	Classifier: RNN, CNN	RNN = 88.89 %	[82]
Continuation of … Parkinson disease detection based on voice dataset			
**Dataset**	**Methodology**	**Accuracy**	**References**
UCI machine learning library and 195 instances and 24 attributes	Classifier: KNN + AdaBoosta.M1, KNN + Bagging, KNN + MLP	KNN + AdaBoosta.M1 = 91.28 %, KNN + Bagging = 90.76 %, KNN + MLP = 91.28 %	[30]
UCI machine learning library	Classifier: SVM, DT, CNN, BiLSTM	SVM = 73.35 %, DT = 73.46 %, CNN = 84.29 %, BiLSTM = 87.48 %	[83]
Multi-Variate vocal data	Classifier: ADNN, ADRNN, ADCNN, SiamesePointNet + PCA + deep clos-est point (SPPD), CNN, RNN	ADNN = 98.90 %, ADRNN = 99.47 %, ADCNN = 99.78 %, SPPD = 98.66 %, CNN = 97.77 %, RNN = 97.82 %	[59]
UCI machine learning library	Classifier: SMOTE, NB, kNN and RF	kNN = 91.45 %, RF = 95.58 %, NB = 84.67 %	[50]
UCI machine learning Repository and PhysioNet Database Bank	Feature extraction: VGFRSD; Classi-fier: VIC ANN	VGFRSD: SVM = 86.12 %, XG-Boost = 78.66 %, MLP = 87.79 %; VIC: SVM = 81.16 %, XGBoost = 77 %, MLP = 85.60 %	[64]
PPMI database	Classifier: Deep Ensemble	96.68 %	[31]
Not Given	Classifier: SVM	91.18 %	[63]
Not Given	Classifier: DNN	85 %	[84]
UCI Machine learning repository	Classifier: SVM, RF, NB, kNN	RF = 90.26 %, NB = 69.23 %	[60]
M.A. Little's Oxford recording	Classifier: ANN	100 %	[85]
UCI Machine learning repository	Classifier: Incremental SVM	Reduce prediction computing time	[41]
PPMI database	Classifier: MLP, BayesNet, RF and BLR	BLR = 97.159 %	[61]
Parkinson Disease data sets	Classifier: SVM, KNN, and LR	SVM = 100 %, LR = 97 %, kNN = 60 %	[64]
UCI Machine learning repository	Classifier: MLAs	C4.5, SVM, and ANN perform better than others.	[67]
UCI Machine learning repository	Classifier: DT, attribute selection mea-sures, ID3 and decision stumps	decision tree algorithm performs best	[68]
Dataset contains 5875 instances and 26 attributes	Classifier: Weka V3.4.10 and Orange V2.0b software	RF = 90.2 %	[60]
44 characteristics were retrieved from five categories.	Classifier: BayesNet	75.2 %	[40]
UCI Machine learning repository	Feature extraction: Cuttlefish algo-rithm; Classifier: DT and kNN classi-fiers	kNN = 92.19 %	[69]
UCI Machine learning repository	Feature extraction: TQWT; Classifier: mRMR and SVM-RBF	SVM-RBF = 86 %	[53]
Not Given	DNN, Motor-UPDRS and Total UP-DRS were tested.	Motor-UPDRS = 81.66 %	[86]
Not Given	Classifier: SVM	92.21 %	[36]
Smartphone (SP) and acoustic car-dioids (AC) audio signals	Classifier: KNN, MLP and SVM	For SP = 94.55 %, For AC = 92.94 %	[44]
Not Given	Feature extraction: ALO algorithm Classifier: KNN, DT, and RF	RF = 95.91 %	[65]
UCI machine learning repository	Feature extraction: Wolf optimization algorithm; Classifier: ANN, RF, and DT	ANN = 93.87 %	[66]
UCI machine learning repository	Feature extraction: SVD and NCA Classifier: kNN, SVM, CNN, RF, NB, DT	CNN = 98.41 %	[52]
UCI machine learning repository	Feature extraction: mRMR and RFE; Classifier: XGboost	RFE + XGboost = 95.39 %	[71]
UCI machine learning repository	Classifier: CNN	86.9 %	[72]
UCI machine learning repository	Feature extraction: SMOTE technique; Classifier: RF	94.8 %	[57]
UCI machine learning repository	kNN, SVM, CNN, RF, NB, DT	RF = 99.49 %	[62]
UCI machine learning repository	Feature extraction: After MAMa tree preprocessing, SVD and relief-based technique were used to pick features; Classifier: kNN	92.46 %	[52]
Spanish language PC-GITA data	Feature extraction: CNN with ALEXNET; Classifier: MLP	99.3 %	[87]
UCI Parkinson's disease classifica-tion data set	Feature extraction: Bat algorithm	96.74 %	[73]
Biomedical record	Feature extraction: Wrapper method; Classifier: RF, MLP, SVM, and KNN	SVM-RBF = 94.7 %	[46]
Not Given	Feature extraction: ReliefF method; Classifier: KNN and SVM	SVM = 91.25 %	[56]
Not Given	Feature extraction: RFE; Classifier: RT, ANN, and SVM	RFE + SVM = 93.84 %	[38]
PCA and OFS based feature sets	Bagging classification, RT (Bagging Classification And Regression Trees (CART)), RF, Recursive PARTitioning (RPART)	RF with PCA = 96.83 %	[37]
Continuation of … Parkinson disease detection based on voice dataset			
**Dataset**	**Methodology**	**Accuracy**	**References**
biomedical voice of human	FCM clustering and pattern recogni-tion methods	68.04 %	[74]
University of Pennsylvania 40-item smell identification test (UPSIT-40)	Classifier: LR	89.0 %	[75]
RBDSQ	Classifier: SVM and classification tree methods	SVM = 85.48 %	[76]
Local field potential signals	RBF, SVM, MLP	SVM = 81.14 %; RBF = 80.13 %; MLP = 79.25 %	[88]
Gait characteristics	Feature extraction: Wavelet-based fea-ture extraction; Classifier: Neural Net- work with weighted fuzzy membership functions	77.33 %	[89]
Gene expressions	Classifier: Independent component analysis (ICA) + Meta-cognitive Neural Network (MCNN)	95.55 %	[90]
Movement disorder	Feature extraction: Wrapper feature selection; Classifiers: NB, kNN, LDA, C4.5 decision trees, ANN	NB = 82.08 %, KNN = 80.06 %, LDA = 83.24 %, C4.5 = 81.50 %, ANN = 64.74 %	[91]
Brain MRI images	Voxel-Based Morphometry + Periph-eral blood lymphocytes (PBL)-meta cognitive radial basis function network (McRBFN) + RFE	87.21 %	[92]
PPMI dataset and Virgendela Vic-toria” Hospital in M'alaga (VV), Spain	ICA + SVM	PPMI dataset = 91.3 % and Virgendela Victoria” Hospital in M'alaga (VV), Spain = 94.7 %	[32]
T1-weighted MRI Images	KSOM + Least Square SVM	99.9 %	[93]
Voice Assessment	PCA + SVM	87.50 %	[94]
Acoustic features extracted from replicated voice recordings (Biomedical)	Gibb's Sampling Algorithm + Bayesian Approach	86.2 %	[40]
UCI machine learning repository	Fuzzy neural system with 10-fold cross validation	100 %	[11]
UCI machine learning repository	Feature extraction: RPART, C4.5, PART, Bagging; Classifier: CART, RF, Boosted C5.0, SVM	SVM = 97.57 %	[12]
UCI machine learning repository	deep belief network (DBN) of 2 re-stricted Boltzmann machines (RBMs)	94 %	[95]
UCI machine learning repository	enhanced fuzzy minmax neural net-work with the OneR attribute evalua- tor (EFMM-OneR) with 10-fold cross validation or 5-fold cross validation	94.21 %	[48]
UCI machine learning repository	Classifier: LR, LDA, Gaussian NB, DT, kNN, SVM-linear, SVM-RBF with Leave One subject out (LOSO) cross validation	SVM = 70 %	[96]
UCI machine learning repository	LDA–NN–GA with LOSO cross valida-tion	95 %	[74]
UCI machine learning repository	Nearest Neighbor Like (NNge) with AdaBoost with 10-fold cross validation	96.30 %	[97]
UCI machine learning repository	Classifier: LR, kNN, NB, SVM, DT, RF, DNN with 10-fold cross validation	kNN = 95.513 %	[98]
UCI machine learning repository	Classifier: MLP with a train-validation-test ratio of 50:20:30	92.96 %	[99]
UCI machine learning repository	Classifier: FKNN, SVM, KELM with 10-fold cross validation	FKNN = 97.89 %	[100]
UCI machine learning repository	Classifier: SVM, LR, GB, RF with train-test split ratio = 80:20	LR = 76.03 %	[101]
UCI machine learning repository	Classifier: MLP, GRNN with a training-test ratio of 50:50	General regression neural network (GRNN) = 99.01 %	[102]
UCI machine learning repository	Classifier: Eosinophil chemotactic fac-tor of anaphylaxis (ECFA)-SVM with 10-fold cross validation	97.95 %	[103]
UCI machine learning repository	Classifier: Fuzzy classifier with 10-fold cross validation, LOO cross validation or a train-test ratio of 70:30	100 %	[104]
UCI machine learning repository	Classifier: Averaged perceptron, boosted DT, locally deep SVM, LR, NN, SVM with 10-fold cross-validation	Boosted DT = 0.912105	[105]
UCI machine learning repository	Classifier: kNN, SVM, Extreme Learn-ing Machines (ELM) with a train vali-dation ratio of 70:30	SVM = 96.43 %	[53]
UCI machine learning repository	Classifier: CNN with LOO cross vali-dation	0.869	[72]
Continuation of … Parkinson disease detection based on voice dataset			
**Dataset**	**Methodology**	**Accuracy**	**References**
UCI machine learning repository	Classifier: SVM, LR, KNN, DNN with a train test ratio of 70:30	DNN = 98 %	[106]
UCI machine learning repository	Classifier: SVM-RBF, SVM-linear with 10-fold cross validation	99 %	[106]
UCI machine learning repository	Classifier: Least Square-SVM, Proba-bilistic Neural Network (PNN), GRNN with conventional (train-test ratio of 50:50) and 10-fold cross validation	LS-SVM or PNN or GRNN = 100 %	[80]
UCI machine learning repository	Classifier: SVM-linear, FBANN with 10-fold cross validation	Feedback ANN (FBANN) = 97.37 %	[16]
UCI machine learning repository	Classifier: SVM-linear with 5-fold cross validation	99.87 %	[107]
UCI machine learning repository	Classifier: DT, RF, SVM, GBM, XG-Boost	SVM-linear: FNR = 72.5 %	[108]
UCI machine learning repository	CART, SVM, ANN	SVM = 93.84 %	[38]
UCI machine learning repository	EWNN with a train-test ratio of 90:10 and cross validation	92.9 %	[109]
UCI machine learning repository	Stacked generalization with Consensus based Matching and Tracking of key- points Neural Network (CMTNN) with 10 fold cross validation	70 %	[110]
UCI machine learning repository	HMM, SVM	HMM = 95.16 %	[111]
UCI machine learning repository	Improved Parallel Grey Wolf Optimiza-tion (IGWO)-KELM with 10 fold cross validation	97.45 %	[107]
UCI machine learning repository	Stochastic Compositional Frank-Wolfe (SCFW) -KELM with 10 fold cross val-idation	99.49 %	[112]
UCI machine learning repository	SVM-RBF with 10-fold cross validation	96.29 %	[113]
UCI machine learning repository	LR, NN, SVM, SMO, Pegasos, Ad-aBoost, ensemble selection, FURIA, rotation forest BayesNet with 10-fold cross-validation	Average accuracy across all models = 97.06 % SMO, Pegasos, or AdaBoost = 98.24 %	[114]
UCI machine learning repository	LR, KNN, SVM, NB, DT, RF, ANN	ANN = 94.87 %	[115]
UCI machine learning repository	KNN	90 %	[116]
UCI machine learning repository	RFE with 10-fold cross validation	87.1 %	[117]
UCI machine learning repository	SVM-RBF with 10-fold cross validation or a train-test ratio of 50:50	98.95 %	[95]
UCI machine learning repository	ELM with 10-fold cross validation	88.72 %	[48]
UCI machine learning repository	Ensemble learning with 10-fold cross validation	90.6 %	[118]
UCI machine learning repository	Generalized Low-Rank Approximation (GLRA), SVM, bagging ensemble with 5-fold cross validation	95.58 %	[31]
UCI machine learning repository	DT classifier, LR, SVM with 10-fold cross validation	SVM = 76 %	[119]
UCI machine learning repository	KNN, SVM with 10-fold cross valida-tion	SVM = 91.25 %	[56]
UCI machine learning repository	Maximum a Posteriori Probability (MAP), SVM-RBF, FLDA with 5-fold cross validation	MAP = 91.8 %	[120]
Collected from participants	SVM (RBF, linear, polynomial, and MLP kernels) with LOSO	SVM-linear = 85 %	[94]
Collected from participants	SVM-RBF with cross validation	81.8 %	[121]
Collected from participants	SVM with stratified 10 fold cross vali-dation or LOO cross validation	94.4 %	[122]
Not Given	KNN, SVM-linear, SVM-RBF with LOSO or SLOO	SVM-linear = 77.50 %	[70]
Not Given	KNN, SVM-linear, SVM-RBF, ANN, DNN with LOO cross validation	SVM-RBF = 84.62 %	[123]
Not Given	KNN, SVM-linear, SVM-RBF, ANN, DNN with LOSO cross validation	SVM-RBF = 89.3 %	[92]
Not Given	RF, SVM with 10-fold cross validation and a train-test ratio of 90:10	SVM = 98.6 %	[35]
] Not Given	RF with internal OOB validation	EER = 19.27 %	[124]
The Neurovoz corpus	Siamese LSTM-based NN with 10-fold cross validation	EER = 1.9 %	[125]
mPower database	L2-regularized LR, RF, gradient boosted DT with 5-fold cross valida-tion	Gradient boosted DT = 90.1 %	[126]
PC-GITA database	ResNet with train validation ratio of 90:10	91.7 %	[127]

**Table 6:** Parkinson disease detection based on hand writings and drawings.

P	arkinson disease detection based on hand	writings and drawings	
**Dataset**	**Methodology**	**Accuracy**	**References**
NewHandPD	RF, LR, SVM	SVM = 89.4 %	[129]
Forty subjects, 20 PD and 20 con-trols (aged 38–81)	Multivariate analysis of variance (MANOVA) analyses were used to test for group differences (controls vs PD)	97.5 %	[23]
Continuatio	n of … Parkinson disease detection based	on hand writings and drawings	
**Dataset**	**Methodology**	**Accuracy**	**References**
PaHaW	RNN, LSTM, BiGRU	LSTM = 89.64 %	[51]
PaHaW	3CNN, SVM, SVM-RBF	3CNN = 86.67 %	[45]
HandPD	CNN	98 %	[25]
Mindwave EEG	OPF, SVM, and Bayesian classifier	SVM = 100 %	[49]
37 PD (19 men/18 women) and 38 age- and gender-matched healthy controls.	SVM with radial Gaussian kernel	88.13 %	[34]
Kaggle handwriting dataset VGG-19	CNN	AlexNet 89 %	[130]
HandPD	Adaboost model	76.44 %	[131]
HandPD + NewHandPD	Deep transfer learning-based algo-rithms	99.22 %	[132]
32 people (21 men, 11 women, 71.4 8.3 years old).	ANN	90 %	[58]
HandPD	CNN	95 %	[25]
PDMultiMC	SVM classifier with RBF kernel	96.875 %	[47]
HandPD	DT and KNN classifiers	92.19 %	[69]
HandPDMultiMC	SVM	96.87	[47]
PaHaW	XGBoost	97.14	[133]
Handcrafted feature	NB	88.6 %	[134]
Handcrafted feature	NB	93.3 %	[135]
Not Given	DESN	89.3 %	[39]
	CNN	72.5 %	[136]
Not Given	CNN	96.5 %	[32]
parkinsonian patients and 73 con-trol; subjects participated in those studies	DNN	98.7 %	[54]
93 patients with idiopathic; PD (59 males and 34 females) and 73 healthy control subjects (40 males and 32 females).	DNN	99.1 %	[55]
The PC-GITA database	LSTM, CNN	91.7 %	[127]

Learning Machines (ELM), Independent Component Analysis (ICA), Prin-cipal Component Analysis (PCA), Self-Concordant Frank-Wolfe (SCFW), Hidden Markov Model (HMM), and others.

Interestingly, some researchers generated their own datasets, extracted features, and then employed ML or DL techniques to predict early PD us-ing digital tools such as smart pens, digitizers, sensor arrays, computerized positron emission tomography (com-PET), and more, as evidenced by works like [[23], [24], [25], [26]], and [27]. However, most researchers relied on hospital and publicly available datasets for their PD diagnosis endeavors.

It's important to note that we excluded studies falling into one or more of the following categories.

• Investigations related to Parkinsonism or associated disorders that did not involve PD classification or diagnosis.
• Studies aimed at distinguishing PD from other forms of tremor-related muscle rigidity.
• Research that did not incorporate metrics quantifying classification per-formance.
• Studies lacking adequate or accurate descriptions of the ML algorithms, datasets, or subjects used in the research.

### 3.2 Selection process

We conducted a rigorous quality evaluation of the included articles in accordance with the guidelines provided by the PRISMA, as illustrated in Fig. 4. These guidelines have been meticulously followed throughout the entire research process, encompassing the search for relevant articles, their screening, and the retrieval of pertinent data. To ensure the inclusion of appropriate research publications, we established clear criteria for eligibility and ineligibility. This systematic approach allowed us to select a diverse range of research papers, which were subsequently downloaded for thorough evaluation and discussion within this review. The review process undertaken for this article comprised several distinct stages, as depicted in the workflow diagram shown in Fig. 3. Each of these stages has been executed metic-ulously to ensure the comprehensive and systematic analysis of the chosen research papers.

#### 3.2.1 Gathering key information for review

The key information extracted from the articles, such as a) Objectives, b) Dataset, c) Methodology, d) Performance, e) Number of Subjects, f) Feature Extraction Method, g) Classifiers, h) Year, and i) Reference.

The year of publishing refers to the year in which the study was initially published online and then archived in another year. This definition applies to studies that were archived in a subsequent year. In the event that this in-formation was not readily available, the year that the article was copyrighted was considered to be the publishing year. In the research that developed new models and only used previously established models for the purpose of com-parison, information pertinent to the newly developed models was gleaned.

#### 3.2.2 Objectives of the study

We have categorized the included studies based on their specific type of diagnosis and overarching objectives. This categorization allows us to delineate the diverse goals and purposes pursued by these investigations. The diagnostic aspects explored in this research can be segmented into two primary components.

a) **Assessment or Diagnosis of PD:** This involves the comparison of data collected from individuals with Parkinson's disease (PD) against data from control participants. The aim is to assess and diagnose PD accurately.
b) **Diagnostic Testing:** This category encompasses studies focused on developing and evaluating diagnostic tests for PD. These tests aim to provide reliable and effective means of diagnosing the condition.

This division helps clarify the multifaceted nature of research objectives within the realm of PD diagnosis.

#### 3.2.3 Evaluation of the model

A comprehensive list of performance metrics utilized for evaluating the ML models discussed in the article is presented in Table 2. While these metrics may be well-known to specialists in the field, they serve as a valu-able resource for swiftly comparing the effectiveness of various models in PD identification. Metrics of performance that are utilized in the process of eval-uating ML and DL models are: (a) Accuracy, (b) Precision, (c) F1-Score, and (d) Recall [28].

### 3.3 Data collection process

**Fig. 5:**
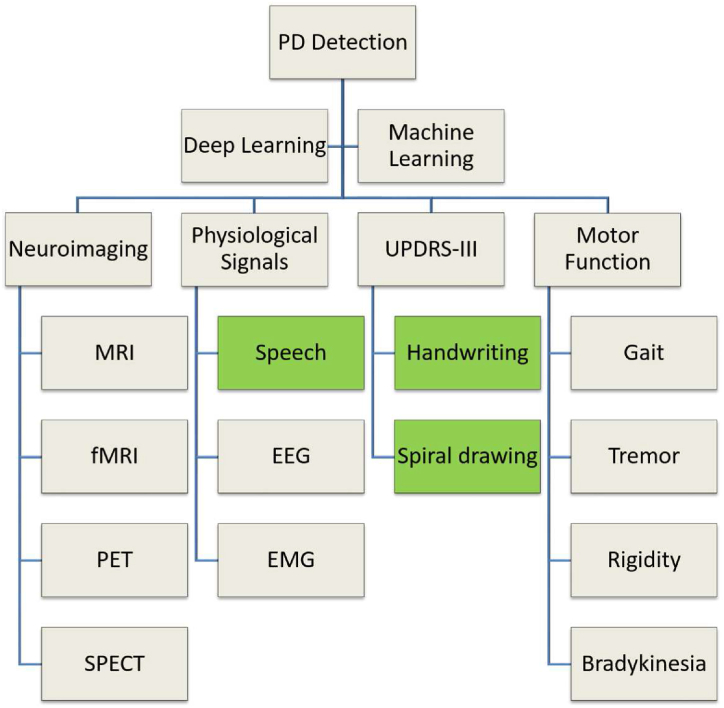
Parkinson's Disease (PD) diagnostic modality categorization.

Fig. 5 visually illustrates the diverse modalities employed in diagnos-ingPD through ML and DL approaches. Notably, this paper focuses on three specific modalities: Speech, Handwriting, and Spiral Drawing, highlighted in green.

**Fig. 6:**
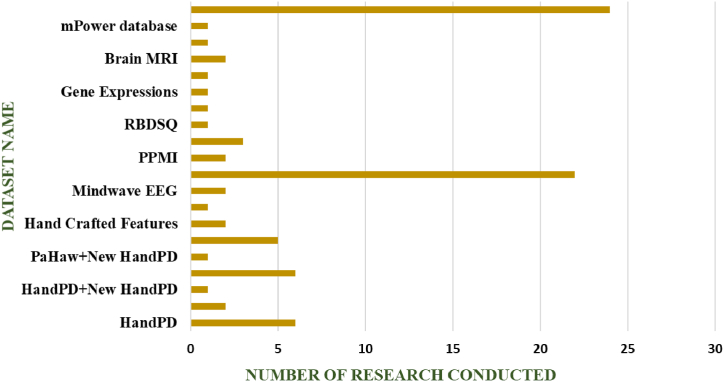
Datasets that are used in various researches.

Fig. 6 provides a graphical representation of the frequency of various datasets used in prior research papers. This visual representation offers in-sights into the prevalence of different datasets within the literature, aiding in understanding the data landscape in PD diagnosis studies.

#### 3.3.1 Parkinson voice dataset

The dataset used in this study was produced by Max Little of Oxford University in collaboration with the National Centre for Voice and Speech in Denver, Colorado [29]. It includes voice measures from 31 participants, 23 of whom had been diagnosed with PD [29]. Each row in the dataset corresponds to one of the 195 voice recordings, with each column representing a different voice measure [29]. To distinguish between healthy individuals and those. with PD, the ”status” column is set to 0 for healthy participants and 1 for those with PD [29]. This dataset is valuable for research focused on PD diagnosis and voice feature extraction.

Additionally [30], utilized data from the UCI machine learning library in their study. This dataset contains 195 instances and 24 attributes. ML algorithms were employed to enhance the dataset's effectiveness and facili-tate early disease detection. The researchers conducted experiments to com-pare different techniques and identify the most accurate one. According to their findings, KNN and ANN exhibited superior precision compared to other methods. Furthermore, combining these two classifiers not only increased ac-curacy but also reduced the time required for model building. Notably, both AdaBoost.M1 and MLP with KNN achieved an impressive accuracy of 91.28 percent, showcasing their potential for PD diagnosis.

#### 3.3.2 PPMI dataset

The Parkinson's Progression Markers Initiative (PPMI) is an extensive research project aimed at investigating experimental therapeutics and epi-demiology related to PD [31,32]. This initiative encompasses individuals diagnosed with PD, individuals at a significant risk of developing PD, as well as healthy individuals [31,32]. With 600 instances, the PPMI dataset is sub-stantial and well-documented. It includes 400 PD patients and 200 healthy controls, making it ideal for comparative studies. This dataset, released in December 2011, offers 159 attributes with numerical and categorical data types. It has been cited extensively in 1345 research papers and is suitable for classification tasks with no missing values.

#### 3.3.3 HandPD

The HandPD dataset [27] comprises handwriting assessments collected from two distinct groups: (i) a healthy group and (ii) a group of Parkinson's patients. In total, there are 18 healthy adults in the Healthy Group and 74 patients in the Patients Group. Among the participants, there are six men and 12 women in the Healthy Group, with ages ranging from 19 to 79 and an average age of 44. Additionally, two individuals in the Healthy Group are left-handed, while 16 are right-handed. In the Patients Group, there are 59 men and 15 women, aged between 38 and 78, with an average age of 58. Within the Patients Group, five individuals are left-handed, and 69 are right-handed. The dataset comprises a total of 736 images, which are further categorized into two groups: healthy (consisting of 72 images) and patient (comprising 296 images). Among the patient images, there are 368 images from each of the two drawing types: spirals and meanders.

#### 3.3.4 mPower database

In March 2015, the mPower study [33] was initiated. It is an observational smartphone-based research endeavor that leverages Apple's ResearchKit li-brary to assess the feasibility of routinely collecting data on Parkinson's disease symptoms and medication sensitivity remotely. The collected data enables the classification of individuals into control groups, PD self-reporters, and the assessment of PD severity. Each data stream within the study presents a myriad of challenges that necessitate solutions from the academic community. The mPower Database is expansive, featuring 898 instances. Unfortunately, details about the number of PD patients are not specified. It is based on app data and contains eight attributes. Released in 2016, this dataset has been cited in 337 papers, making it valuable for classification tasks. No missing values are reported.

#### 3.3.5 PaHaw dataset

38 age- and gender-matched healthy controls and 37 people with PD patients' handwriting data are collected by PaHaW [34]. Participants were accepted at Masaryk University's First Department of Neurology and St. Anne's University Hospital in Brno, Czech Republic. The participants were right-handed and fluent speakers of Czech. There were no variations in gender or age. None of the subjects had any central nervous system disorders, with the exception of Parkinsonism. Prior to acquisition, patients were exclusively evaluated in the ON-state while taking dopaminergic drugs. It was also determined that no mobility issues or injuries affected handwriting in the healthy control (HC) group. PaHaw comprises 75 instances, with 37 PD patients and 38 healthy controls. This dataset focuses on handwriting data and contains 16 attributes. Released in 2016, it has garnered 290 citations, indicating its utility for classification tasks. No missing values are reported.

#### 3.3.6 NewHandPD

The NewHandPD dataset [25] is an extension of the HandPD dataset. It incorporates images from two drawing activities: the standard spiral cogni-tive test and the modified spiral (meander) test. Additionally, NewHandPD includes both offline images and online signals, which are time-based se-quences. Handwriting signals were recorded using a Biometric Smart Pen (BiSP). This dataset comprises data from 31 patients and 35 healthy indi-viduals, with a participant breakdown of 39 men and 29 women, most of whom are right-handed (59 out of 66 participants). Similar to HandPD, the NewHandPD dataset was released in 2016 and contains a total of 264 instances. It consists of 31 PD patients and 35 healthy controls, although specific attribute information is not provided. Like HandPD, this dataset is suitable for classification tasks but may require further exploration to fully realize its potential.

#### 3.3.7 PDMultiMC

The PDMultiMC dataset [35] comprises a total of 32 individuals, evenly split between PD patients and healthy controls. Among the participants, there are 16 PD patients, including 12 males and 4 females, selected from the University of Balamand Medical Center and Saint George Hospital in Beirut, Lebanon. The control group consists of 16 individuals, composed of 5 males and 11 females, who are not only healthy but also carefully matched in terms of handedness, age, and education. The dataset provides a range of information for each subject, including age, gender, years of education, disease stage measured by the Hoehn and Yahr scale, daily Levodopa dose, Unified PD Rating Scale (UPDRS) scores for Part I, II, and III, a brief mental state assessment, and disease duration. PDMultiMC is the smallest among the datasets discussed, containing 32 instances, with an equal distri- bution of PD patients and healthy controls. Released in 2018, it encompasses 16 attributes related to demographic information. While it may not be as extensive as some other datasets, it offers a unique perspective on PD diag-nosis. However, it is less commonly utilized for classification tasks, with only 32 citations reported. Importantly, no missing values have been reported in this dataset (see Table 3).

### 3.4 Features analysis

Feature selection is a dimensionality reduction strategy used in medical diagnosis problems that seeks to pick a small subset of the relevant charac-teristics from the original features by eliminating duplicate, unnecessary, or noisy qualities. The features employed in our research for both the speech and text datasets are shown in Table 4.

### 3.5 Review based on UCI datasets

#### 3.5.1 Machine learning approaches

In a study by Lamba et al. (2022) [50], a hybrid speech-based tech-nique for PD diagnosis was proposed. The research involved experimenting with various feature selection techniques and classification algorithms to cre-ate an optimal model. They explored combinations of three feature selec-tion methods—mutual information gain, extra trees, and evolutionary algo-rithms—and three classifiers—NB, kNN, and RF. The study utilized a speech dataset from UCI's machine learning repository and addressed class imbal-ance with Synthetic Minority Oversampling Technique (SMOTE). The ge-netic algorithm coupled with an RF classifier achieved an accuracy of 95.58 %. Additionally, using smaller feature subsets from feature selection improved the performance of all three classifiers, with genetic algorithms outperform-ing previous methods and yielding a significant 21.70 % improvement. Future work aims to validate this approach on larger speech and voice datasets, con-sidering that early PD symptoms, such as slowness and tremors, can affect handwriting.

In the study by Srieam et al. (2015) [60], it was demonstrated that the PD dataset exhibits more parallel dimensions. SVM achieved the highest accuracy at 88.9 %, surpassing majority voting and KNN. RF achieved an accuracy of 90.26 %, while NB lagged behind at 69.23 %. Hierarchical clus-tering and self-organizing maps (SOM) were used for prediction, indicating more clusters in healthy datasets and fewer in diseased datasets.

Nayan et al. (2016) [61] expanded upon a previous study that incor-porated rapid eye movement sleep behavior disorder and olfactory loss as biomarkers for PD diagnosis. This research applied new ML techniques, including MLP, Bayesian Network (BayesNet), RF, and boosted LR. The boosted LR model achieved the highest accuracy at 97.159 % and an impres-sive receiver operator characteristic score of 98.9 %, suggesting the potential for early PD prediction using these models.

Senturk et al. (2020) [38] presented a machine learning-based approach to PD diagnosis, employing feature selection and categorization. Feature selec-tion methods included feature importance and recursive feature elimination. The study explored classification trees, Neural Networks (NNs), and SVMs, with SVMs utilizing a Rotation Forest Ensemble (RFE) outperforming other approaches. The resulting PD diagnosis achieved an accuracy of 93.84 %, with a focus on vocal traits.

Polat et al. (2012) [57] proposed the use of speech signals for PD diag-nosis. They employed SMOTE preprocessing on the dataset. Utilizing a RF classifier, they achieved an impressive accuracy of 94.8 %.

In the study by Mostafa et al. (2020) [62], the diagnosis of PD focused on evaluating vocal problems. The researchers applied feature selection tech-niques employing an innovative multiple-feature evaluation method. Five different classifiers were used to examine the reduced feature subset, with the RF classifier outperforming the rest, achieving a remarkable accuracy of 99.49 %.

Tuncer et al. (2020) [52] employed vowel analysis to detect PD. They uti-lized preprocessing techniques including Minimum Average Maximum (MAMa) tree pre-processing and feature selection through singular value decomposi-tion and relief-based methods. The study incorporated eight different classi-fiers, with the KNN classifier achieving an accuracy of 92.46 % for PD diag-nosis.

Soumaya et al. (2019) [63] employed optimization techniques, includ-ing the genetic algorithm (GA), to enhance the performance of SVM for PD diagnosis. They utilized supervised ML to construct classifiers based on models representing various relevant data types. Their proposed classifica-tion model utilized features such as linear predictive encoding (LPC), energy, zero-crossing rate (ZCR), Mel-frequency cepstral coefficients (MFCC), and wavelet Shannon entropy extracted from approximation a3 of the discrete wavelet transform (DWT). With the combination of GA and SVM, they achieved an impressive accuracy of 91.18 %.

Nilashi et al. (2016) [41] developed a predictive model for the Unified PD Rating Scale (UPDRS) using an incremental SVM. They predicted both total-UPDRS and motor-UPDRS scores. The study also involved dimen-sionality reduction through non-linear iterative partial least squares and the generation of self-organizing map clusters. The mean absolute errors (MAEs) for total-UPDRS and motor-UPDRS were 0.4656 and 0.4967, respectively. Their results demonstrated the effectiveness of the proposed technique for UPDRS prediction, which has potential applications in healthcare.

Shamrat et al. (2019) [64] employed AI techniques to detect PD using various datasets. They utilized SVM, KNN, and LR for PD prediction. The classifiers were evaluated based on recall, precision, F1-score, and accuracy. SVM exhibited outstanding performance, achieving 100 % accuracy in PD prediction, while LR achieved an accuracy of 97 %. On the other hand, KNN had a lower precision rate of 60 % for PD datasets. The study highlighted SVM as a robust classifier for analyzing PD datasets and showcased the potential of ML in clinical research.

Lahmiri et al. (2019) [36] focused on diagnosing PD by identifying voice problem patterns. They employed eight pattern ranking algorithms and a nonlinear SVM classifier to differentiate between individuals with PD and healthy individuals. Their approach achieved an accuracy of 92.21 %.

Almeida et al. (2019) [44] proposed a method that leveraged pronuncia-tion and voice to detect PD. They collected audio signals using smartphones and acoustic cardioid devices and then separated the signals into voiced and unvoiced files using specialized software. Feature engineering was employed to extract 144 relevant features, and KNN, MLP, and SVM classifiers were trained using the OpenCV 2.49 Toolbox. The results indicated that pronun-ciation challenges were more accurate than speech tasks, with acoustic car-dioid and smartphone channels achieving accuracies of 94.55 % and 92.94 %, respectively.

Sharma et al. (2019) [65] developed a PD prediction model based on the Ant Lion Optimizer (ALO) algorithm. They used the reduced feature subset generated by the ALO algorithm as input for KNN, decision trees (DT), and RF classifiers, achieving an accuracy of 95.91 %.

Sundaram et al. (2019) [66] proposed a PD detection system using datasets from the UCI machine learning repository. They improved feature selection by updating the Grey-Wolf algorithm and employed KNN, RF, and DT classifiers. Their approach achieved an accuracy of 93.87 % on the speech dataset.

Almeida et al. (2019) [44] conducted an evaluation of their method using various metrics, including the equal error rate (EER), the area under the ROC curve (AUC) values derived from the detection error tradeoff (DET) and ROC curves, accuracy, specificity, and sensitivity. They used these met-rics to assess the classification performance of their approach and compared it to other methods on the same dataset. Their findings revealed that phona-tion was significantly more effective than speech in detecting PD. Using the acoustic cardioid channel, they achieved an accuracy of 94.55 %, an AUC of 0.87, and an EER of 19.01 %. With the smartphone channel, they achieved 92.94 % accuracy, a 0.92 AUC, and a 14.15 % EER.

Shamli et al. (2016) [67] proposed a multi-classifier system based on big data to enhance predictive performance and reduce the time to cost-effective actions. They discussed the concepts of big data and its application in healthcare, including descriptive, predictive, and prescriptive analytics. The study focused on the role of dopamine, a neurotransmitter linked to motor function, and its depletion in PD. They utilized the PD voice dataset from the UCI machine learning library for their analysis. Various predictive models were implemented on the dataset, resulting in multiple classifier ac-curacies. Among the classifiers tested, C4.5, SVM, and ANN performed the best, and the authors selected the top-performing classifier after comparing their results. This approach facilitates efficient analysis of large datasets by organizations.

Azad et al. (2013) [68] developed a PD prediction model based on DT. They provided a comprehensive overview of PD, including its symptoms, consequences, and risk factors. The study employed data mining classifi-cation techniques, such as DTs, attribute selection measures, ID3, and de-cision stumps, on a dataset consisting of 197 instances collected from 31 individuals, sourced from the UCI repository. Performance evaluation was conducted using accuracy and classification error metrics, with 10-fold cross-validation providing unbiased validation results. The experimental results demonstrated that the decision tree method outperformed other techniques in terms of accuracy and classification error.

Naranjo et al. (2019) [40] developed a clinical expert system for PD. They collected voice recordings from 80 participants, including half with PD, who sustained a vowel sound for at least 5 s. By employing methods like waveform matching, they extracted 240 rows and 44 columns of data across five distinct categories. A subject-based Bayesian classification method was used, considering each participant's three sound recordings. Cross-validation resulted in an accuracy of 75.2 %.

Gupta et al. (2018) [69] conducted PD diagnosis using speech, voice, and HandPD datasets. They improved accuracy by reducing the number of features and enhancing the Cuttlefish algorithm. Their proposed system achieved an accuracy of 92.19 %.

Sarkar et al. (2019) [70] proposed a PD classification scheme using speech processing algorithms and ML classifiers. They employed the Tunable Q-factor wavelet transform (TQWT) to extract voice features and used mRMR for feature selection. Six classifiers were applied, and the combination of mRMR and SVM-RBF achieved an accuracy of 86 %.

Tuncer et al. (2020) [52] introduced an approach for gender identification and PD diagnosis based on octopus-based methods. Singular value decompo-sition (SVD) was employed for feature extraction, and neighborhood compo-nent analysis (NCA) was used for feature selection. The approach achieved high accuracy for gender identification (99.21 %), PD diagnosis (98.41 %), and the combined task of PD and gender identification (97.62 %).

Nissar et al. (2021) [71] proposed a voice-based PD detection method. They employed feature selection techniques such as Maximum Relevance — Minimum Redundancy (mRMR) and Recursive Feature Elimination (RFE) and tested eight different classifiers. The combination of RFE and Ex-treme Gradient Boosting (XGboost) outperformed others with an accuracy of 95.39 %.

Gunduz et al. (2019) [72] introduced a PD classification approach based on CNN and utilized UCI speech data. They combined features and models to achieve an overall model-level accuracy of 86.9 %.

Olivares et al. (2012) [73] developed a PD diagnosis system utilizing BAT and the PD categorization dataset from UCI. They fed 23 features into the model's input layer and achieved an accuracy of 96.74 % with a 3.27 % loss.

Solana et al. (2021) [46] proposed a vocal-based pre-diagnosis method for PD. They employed RF, MLP, SVM, and KNN classifiers for feature selection and classification. The SVM-RBF classifier achieved an accuracy of 94.7 %.

Yaman et al. (2020) [56] used vowels to detect PD. ReliefF was used to select acoustic characteristics from the dataset, and KNN and SVM classifiers were employed for classification. The SVM classifier achieved an accuracy of 91.25 %.

Senturk et al. (2020) [38] demonstrated an ML-based PD diagnosis sys-tem using selected features, RFE, and feature importance. They applied regression trees, ANNs, and SVMs, achieving an accuracy of 93.8 % with the RFE and SVM classifier.

Aich et al. (2019) [12] focused on classifying the PD group using PCA and online feature selection based on regression (OFS) non-linear characteristics with a dataset from Max Little University, Oxford. They employed nonlinear classifiers, bagging classification, regression trees, RF, and Recursive Parti-tioning And Regression Trees (RPART) and achieved 96.83 % accuracy using RF with PCA.

Rustempasic et al. (2013) [74] emphasized biomedical voice analysis for PD prediction. They utilized fuzzy c-means (FCM) clustering and pattern recognition to predict PD based on patients’ voices and achieved 68.04 % accuracy, 75.34 % sensitivity, and 45.83 % specificity.

Silveira et al. (2008) [75] conducted a study on Brazilians using the Pennsylvania Smell Identification Test (UPSIT)-40 and Sniffin's Sticks 16- item scent tests. Logistic regression was applied for each feature. Sniffin Sticks achieved 89.0 % specificity and 81.1 % sensitivity, while UPSIT-40 had 83.5 % specificity and 82.1 % sensitivity.

Prashanth et al. (2014) [76] employed olfactory loss from the 40-item UPSIT and sleep behavior disorder from the Sleep Behaviour Disorder ques-tionnaire. SVMs and classification trees were used for training, resulting in an accuracy of 85.4 % and sensitivity of 90.5 %.

Anju et al. (2020) [43] demonstrated that PD can be diagnosed using cell phones to track patients' steps. They suggested the use of a deep MLP clas-sifier that doesn't require communication with doctors and can be based on the affected person's voice and movements. PD patients were identified us-ing missing smartphone data, and 17 studies employing ML for PD detection were reviewed.

Tarigoppula et al. (2018) [77] compared NB, RF, LR, and SVM to detect PD. SVM outperformed NB and RF with an accuracy of 88.9 % for Parkin-son's identification, while LR achieved a respectable accuracy of 83.66 %. SVM and Latent Dirichlet Allocation (LDA) were identified as sensitive clas-sifiers.

Chen et al. (2005) [78] proposed a fuzzy-based KNN model for predicting PD and achieved the best accuracy of 96.07 % using 10-fold cross-validation. Jiang et al. (2016) [79] developed a hybrid model for PD detection, which showed remarkable accuracy in a 10-fold cross-validation study, with the highest precision at 96.47 % and an overall accuracy of 95.97 %.

Hariharan et al. (2014) [80] demonstrated that feature preprocessing can achieve 100 % classification accuracy for the Parkinson's dataset in their experimental results.

#### 3.5.2 Deep learning approaches

In their efforts to aid early disease detection, Johri et al. (2019) [128] de-vised two NNs-based models: the Vascular Endothelial Growth Factor Spec-trogram Detector (VGFRSD) and the Voice Impairment Classifier (VIC). These models harnessed deep dense ANNs for analyzing speech recordings and transforming large-scale gait data into spectrogram images to predict PD. Impressively, the experimental results demonstrated the superior accu-racy of these models, with VGFRSD achieving a classification accuracy of 88.1 %, and VIC achieving 89.15 %. To further enhance detection efficiency and accuracy in the future, the authors propose combining the results of these two modules. This integration aims to incorporate critical features like olfactory sound loss and handwriting distortion, utilizing DL techniques to address the limitations observed in previous studies. Additionally, the au-thors intend to develop new algorithms to streamline processing and lighten the system's computational load.

Wroge et al. (2018) [84] undertook a similar quest, using a dataset consist-ing of both PD and non-PD voice recordings. Their research focused on eval-uating how effectively supervised classification techniques, including DNN, could identify diseases. Their findings were noteworthy, as they achieved a remarkable accuracy rate of 85 %. This performance is especially significant, as it implies the potential for non-experts (73.8 %) and movement disorders specialists (79.6 %, rising to 83.96 % after follow-up) to make highly accurate medical diagnoses.

Sadek et al. (2019) [85] contributed to the field by presenting a NN system that incorporates backpropagation to assist clinicians in recognizing PD. Their NN design aimed to improve the robustness of PD prediction, achieving a remarkable network recognition rate of 100 %. While prior re-search had demonstrated 93 % accuracy in predicting PD, Sadek et al. (2019) [85]'s innovative approach showcased substantial advancements, particularly in handling smaller classes and ensuring reliable diagnosis.

Quan et al. (2021) [83] focused on static and dynamic elements of speech in PD diagnosis. They observed differences in the trajectory of the funda-mental frequency curve and articulation alterations between healthy individ-uals (HC speakers) and PD patients. To enhance PD identification, they proposed using BiLSTM models on time-series dynamic speech data. These models quantified dynamic speech properties by analyzing the energy con-tent of voiced-to-unvoiced transitions (onset) and voiced-to-voiced transitions (offset). Their approach outperformed existing ML models that rely solely on static features, leading to improved PD detection accuracy.

Nagasub Rahman et al. (2021) [59] conducted deep multivariate vocal data analysis (DMVDA) using various DNN architectures, including acoustic DNN, acoustic deep RNN, and acoustic deep CNN. DMVDA employed a multivariate approach to process voice attributes and improved acoustic data sampling. This study introduced DL algorithms for analyzing heterogeneous datasets to identify Parkinson's symptoms, resulting in a 3 % performance improvement.

Martinez et al. (2018) [32] employed CNN to detect PD based on sketch-ing movements. Their CNN architecture included feature extraction through convolutional layers and classification using fully connected layers. By an-alyzing PD spiral drawings from digital graphics tablets, their approach achieved impressive results with 96.5 % accuracy, a 97.7 % F1-score, and a 99.2 % AUC, showcasing the potential of CNNs in PD diagnosis.

Wodzinski et al. (2019) [127] used vowels with sustained phonation and a Residual Networks (ResNet) architecture for picture classification to detect PD. They estimated the audio spectrum and fed it into a ResNet architecture, achieving a validation set accuracy above 90 %. This approach demonstrated the transferability of natural image attributes to artificial images representing voice spectrograms.

Alissa et al. (2022) [82] explored the use of DL, RNN, and CNN to dif-ferentiate between healthy individuals and PD patients, considering multiple datasets, including imaging and movement data. They aimed to determine which PD test, whether imaging or time series data, is more effective for diagnosis.

Oh et al. (2020) [81] proposed a CNN-based system for automatic PD detection using EEG signals. They utilized 20 PD and 20 normal EEG sig-nals, achieving an accuracy of 88.25 %, sensitivity of 84.7 %, and specificity of 91.77 %, showcasing the potential of CNNs in diagnosing brain abnormalities associated with PD.

Wu et al. (2017) [31] compared various ML and ensemble learning tech-niques on a dataset containing 183 healthy individuals and 401 early-onset PD patients. Their suggested model achieved an average accuracy of 96.45 %. The ensemble network outperformed other methods, achieving 96.68 % accu-racy and a balanced trade-off between sensitivity and specificity.

Grover et al. (2018) [86] proposed a method to predict PD severity us-ing DNN and UPDRS scores, with motor-UPDRS achieving an accuracy of 81.66 %, higher than total-UPDRS.

Zahid et al. (2020) [87] introduced a spectrogram-based technique for PD diagnosis using Spanish PC-GITA data. They explored three methods, achieving an impressive 99.3 % accuracy with a MLP classifier.

### 3.6 Review based on handwritings related dataset

Pereira et al. (2016) [25] developed a PD detection model focused on impaired writing skills. They proposed a method to learn pen-based features from smart pen signals using ImageNet and LeNet CNN architectures. For meanders and spirals, using ImageNet and Optimum Path Forest (OPF) achieved the highest accuracy at 83.77 %. This approach leveraged pen-based signals to identify PD-related impairments in writing.

Taleb et al. (2017) [47] employed CNN and CNN-BiLSTM models for time series classification to detect PD. Instead of using raw time series data, they encoded pen-based signals into spectrograms, which were then processed as CNN images. These models were trained on extensive datasets and em-ployed various data augmentation techniques for pen-based signals. Among their experiments, CNN-BiLSTM models trained with jittering and artificial data augmentation demonstrated the highest accuracy (97.62 %) for early PD diagnosis. This study highlighted the effectiveness of DL models even with limited data and outperformed pre-engineered models.

Xu et al. (2020) [129] took a different approach by combining RF clas-sifiers and PCA to distinguish PD patients from HC based on handwriting data. They constructed six separate RF models for different handwritten exams to generate class probability vectors representing individual category predictions. A voting method was used to determine the final prediction for each person. Through stratified k-fold cross-validation, their ensemble model showcased superior performance compared to single RF-based strate-gies across six different handwritten tests. The RF ensemble model achieved promising accuracy (89.4 %), specificity (93.7 %), sensitivity (84.5 %), and an F1-score (87.7 %) when evaluating multiple handwriting assessments. This approach outperformed traditional machine learning methods like LR and SVM in classification outcomes.

Drotar et al. (2016) [34] utilized a database containing handwriting sam-ples from 38 HC individuals and 37 PD patients. Participants were asked to write simple phrases and syllables, as well as draw an Archimedean spiral, followed by writing a sentence. The study focused on analyzing new pres-sure features based on the pressure applied to the writing surface. Three classifiers KNN, ensemble Adaptive Boosting (AdaBoost), and SVM—were employed to differentiate between PD patients and HC. SVM showed the best performance in classifying PD using handwriting kinematics and pres-sure, achieving an accuracy of 81.3 % (sensitivity: 87.4 %; specificity: 80.9 %). Pressure features alone contributed significantly to PD diagnosis, with an accuracy of 82.5 %, compared to 75.4 % when using kinematic features.

Shaban et al. (2020) [130] focused on a trained CNN model validated through 4-fold and 10-fold cross-validation. The CNN model exhibited an accuracy of 88 %, 89 %, and sensitivity of 89 %, 87 % when subjected to 10- fold cross-validation. Their proposed approach offered a promising option for diagnosing and screening PD based on handwriting patterns, demonstrating strong performance on two different handwriting datasets compared to a fine-tuned AlexNet.

Ali et al. (2019) [131] suggested a cascading approach to enhance PD de-tection accuracy by combining an Adaboost model with a Chi2 model. The.

Adaboost model was used to predict PD based on a subset of features, result-ing in improved accuracy by 3.3 % and reduced complexity. The cascaded sys-tem achieved an accuracy, sensitivity, and specificity of 76.4 %, respectively, demonstrating promising results.

Kamran et al. (2021) [132] leveraged handwriting samples for early PD diagnosis. Their approach involved combining multiple PD handwrit-ing datasets and employing deep transfer learning-based techniques, which significantly improved performance. The methodology achieved an impres-sive 99.22 % accuracy across various datasets, showcasing its superiority over existing state-of-the-art methods.

Cascarano et al. (2019) [58] conducted a study involving 21 PD patients and 11 HC individuals. Participants were asked to sketch patterns on a graphic tablet while wearing the Myo Armband to quantify forearm muscle activity. Features were extracted from written patterns, pen pressure, move-ment, and muscle activations. These features were used to classify HC versus PD patients and distinguish mild PD from moderate PD using an ANN. The proposed method achieved over 90 % accuracy in detecting and classifying mild and severe PD.

Pereira et al. (2016) [25] introduced a CNN-based approach to extract fea-tures from handwritten dynamics images containing evaluation results. Their method also provided an image- and signal-based dataset for computer-aided PD diagnosis research. This approach outperformed raw data and texture-based descriptors, achieving an accuracy of 95 % in early-stage detection.

Kotsavasi et al. (2017) [134] employed a pen-and-tablet device to analyze hand movements and muscular coordination in healthy individuals and PD patients. They used various metrics related to horizontal velocity and signal entropies. The best classification method achieved an accuracy of 88.63 % and an AUC of 93.1 % in distinguishing between the two groups.

Bernardo et al. (2019) [49] examined patient medical history and clinical exams, including specialized software for drawing specific images. Multiple image algorithms were applied to the drawings to extract 11 metrics from each design. ML algorithms such as OPF, SVM, and NB were used to search for and learn about PD and HC features.

Diaz et al. (2019) [45] explored the concept of ”dynamically improved” static handwriting images. They synthesized enhanced images by combining static and dynamic handwriting features, embedding dynamic information into static representations. The approach outperformed static and dynamic handwriting analysis independently, showcasing the effectiveness of incorpo-rating temporal and velocity information.

Moetesum et al. (2019) [51] presented an approach based on Bidirectional Gated Recurrent Units (BiGRU) to assess handwriting-based sequential in-formation for identifying Parkinsonian symptoms. The resulting feature se-quences were used to train the BiGRU model for prediction, demonstrating the potential of the proposed approach in identifying PD symptoms.

## 4 Result and discussion

**Fig. 7:**
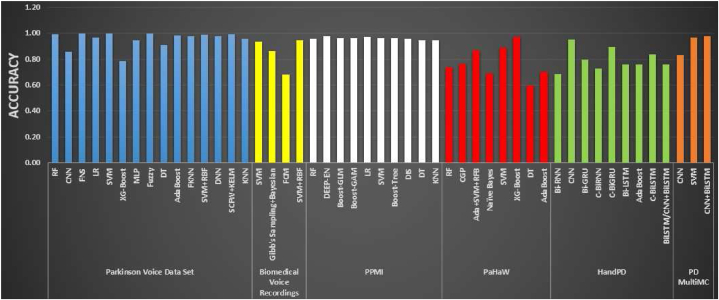
Overall performance conducted in various researches.

Fig. 7 illustrates a comparison of the overall accuracy achieved with various ML algorithms for predicting PD using the most commonly employed dataset. The accuracy of these models can be influenced by several factors, including the characteristics of the datasets used, the number and extraction methods of features, data pre-processing techniques, and the choice of the classifier employed in the final model. Additionally, whether the model is hybrid or not, and whether feature selection techniques are applied, can also impact the accuracy of these predictive models.

In Fig. 7, three prominent ML techniques, namely SVM, Fast Non-dominated Sort (FNS), and fuzzy classifier, achieved the highest accuracy of 100 %, while the XG-Boost algorithm achieved the lowest accuracy of 78.66 %. These results were obtained when using the VGFRSD and VIC techniques on the Physio-Net Database and UCI ML Repository dataset. The AI-based.

SVM and LR models yielded the best results for the PD dataset, with accu-racies of 100 % and 97 %, respectively. The UCI machine learning repository dataset was utilized for FNS and fuzzy classification, both achieving high ac-curacy rates of 99.49 % with 10-fold cross-validation. RF employed a multiple-feature evaluation method to reduce features from the voice disorder dataset and achieved an accuracy of 99.49 %. Without 10-fold cross-validation, the UCI machine learning repository datasets yielded accuracy rates of 86.9 % for CNN, 98 % for DNN, and 91.12 % for DT.

For the Biomedical Voice Recordings dataset, the highest accuracy ob-tained was 94.7 % using SVM-RBF, while the lowest accuracy was 68.04 % with Fuzzy C-Means (FCM). The wrapper method was implemented to en-hance accuracy, achieving 93.84 % when SVM-RBF was used exclusively on data from the National Centre for Voice and Speech in Denver, Colorado. However, the accuracy dropped to 68.04 % when FCM clustering and pattern recognition techniques were applied to biological voice data.

In the PDMultiMC dataset, CNN-BLSTM achieved the best early PD detection accuracy of 97.62 %, while CNN had the lowest accuracy at 83.33 %. Various methods, including SVM, DT, and XG-Boost, were tested on handwriting samples from the PaHaW dataset for early PD diagnosis. Ex-treme Gradient Boosting (XG-Boost) yielded the highest accuracy at 97.14 %, while DT had the lowest accuracy at 59.97 %. Other learning methods for the PaHaW dataset, such as SVM, CGP, AdaBoost, KNN, NB, and RF, demon-strated improved performance, making them state-of-the-art methods.

Using the HandPD dataset, CNN achieved an accuracy of 95.0 %, while bi-directional RNN (BiRNN) reached 68.35 %. Adaptive boosting (AdaBoost) achieved 76.14 % accuracy by selecting crucial features from the feature space. Other algorithms, including CNN-BiRNN, Bi-LSTM, Bi-GRU, and CNN- BiGRU, achieved accuracies of 73.03 %, 76.07 %, 79.03 %, and 89.64 %, respec-tively.

For the PPMI dataset, DL approaches demonstrated substantial improve-ments in accuracy for differentiating PD patients, with an ensemble net-work achieving the highest accuracy of 97.68 % in Fig. 7. BOOST-GAM, BOOST-GLM, and BOOST-TREE closely followed DL with accuracy levels of nearly 96 %. Additionally, RF, LR, SVM, and KNN all achieved good accuracy of over 94.0 % for early PD disease prediction.

### 4.1 Review based on UCI dataset

In the EEG dataset, a CNN achieved notable results with an accu-racy of 88.25 %. However, a slight improvement of 0.64 % was observed when both RNN and CNN were used in the time series dataset. When the Spanish-language PC-GITA dataset was employed along with both CNN and ALEXNET, the approaches achieved an impressive accuracy rate of 99.3 %. For the UCI dataset, KNN and AdaBoost were applied, resulting in an accuracy of 91.28 %. However, when RF was applied to the same dataset, the outcome improved by 4.3 % compared to the previous techniques.

In the T1-weighted MRI images, the combination of Least Squares SVM with Kohonen Self-Organizing Maps (KSOM) achieved an outstanding accu-racy of 99.9 %.

In the multi-variate vocal dataset, the accuracy of Acoustic Deep CNN (ADCNN), Acoustic Deep RNN (ADRNN), and Acoustic DNN (ADNN) reached 99.78 %, 99.47 %, and 98.90 %, respectively.

Finally, in various PD datasets, SVM, ANN on M.A. Little's Oxford recording dataset, and Fuzzy Neural Systems on the UCI machine learning repository dataset all achieved perfect accuracy, with 100 % of the expected results.

#### 4.1.1 Same dataset different methods

**Fig. 8:**
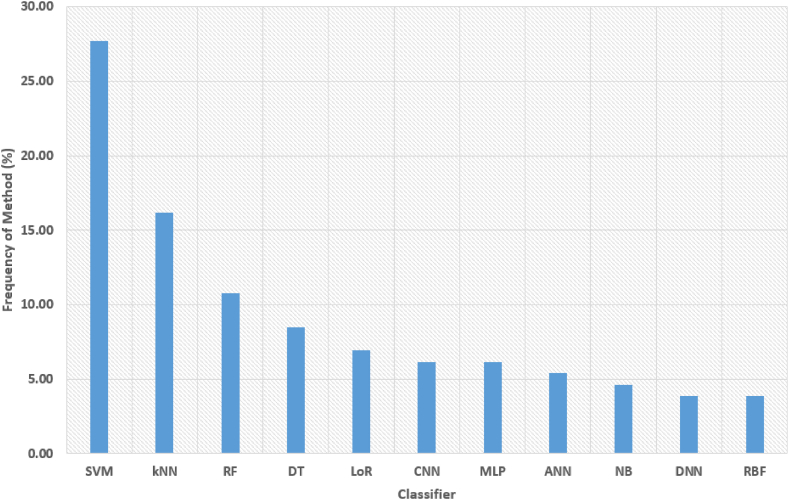
Number of methods (in %) used in UCI dataset.

Many researchers have utilized the UCI voice dataset as a common choice for implementing their algorithms and detecting PD. Over the years, numer-ous ML and DL pipelines have been proposed, often incorporating differ-ent feature selection methods and various algorithms. Fig. 8 provides an overview of this scenario, highlighting that the SVM method has been fre-quently employed in various research papers for PD detection using voice data.

**Hybrid Speech-Based PD Diagnosis:** Lamba et al. (2022) [50] pro-posed a hybrid speech-based PD diagnosis approach using the UCI voice dataset. They experimented with different feature selection techniques and classification algorithms, achieving the highest classification accuracy of 95.58 % using RF.

**Neural Network-Based Models (VGFRSD and VIC):** Johri et al. (2019) [128] developed two neural network-based models, the VGFRSD and VIC, to aid in early disease identification. These models utilized deep, dense ANNs on voice recordings and gait data converted to spectrogram images.

The VIC achieved a classification accuracy of 89.15 %, while the VGFRSD achieved 88.1 % accuracy on the UCI dataset.

**Feature Relevance Analysis with ML Classifiers:** Tarigoppula et al. (2018) [77] proposed a method that involves feature relevance analysis and various ML classifiers. The SVM achieved an accuracy of 88.9 %, and RF achieved an accuracy of 90.26 %.

**Speech Processing and ML Classifiers:** Sarkar et al. (2016) [53] developed a classification system for PD using speech processing methods and ML classifiers. Voice features were extracted using TQWT, and six classifiers were employed. SVM-RBF with feature selection achieved an accuracy of 86 %.

**Vowel Analysis for PD Identification:** Tuncer et al. (2020) [52] used vowel analysis to identify PD. They applied Singular Value Decomposition and relief-based feature selection after preprocessing. The KNN classifier achieved an accuracy of 92.46 %.

**Diagnosis Using BAT (Bayesian Analysis Toolkit):** Olivares et al. (2012) [73] proposed a PD diagnosis technique using BAT. They used 23 features from the UCI PD classification dataset, and their method achieved a 3.27 % loss and 96.74 % accuracy.

**AI-Based PD Prediction:** Shamrat et al. (2019) [64] employed AI to predict PD using various datasets. They used SVM, kNN, and LR classifiers. The SVM achieved 100 % accuracy in PD prediction, while LR achieved 97 % accuracy. However, KNN had the lowest precision (60 %) for PD-related datasets.

#### 4.1.2 Same algorithm different datasets

**Multi-Variate Vocal Data Analysis (VMVDA):** Nagasubramanian et al. (2021) [59] conducted VMVDA on multi-variate vocal data using three different approaches: ADNN, ADRNN, and ADCNN. Their work focused on extracting valuable information from vocal attributes and processing this data using DL algorithms. By incorporating these techniques, they achieved a 3 % improvement in performance for identifying Parkinson's symptoms.

**Parkinson's Diagnosis with ML Methods:** Nayan et al. (2016) [61] utilized the PPMI dataset to diagnose PD. They employed ML methods such as MLP, BayesNet, RF, and boosted LR to build automated diagnostic models. Among these, the boosted LR model achieved the highest accuracy of 97.159 % and an ROC (Receiver Operating Characteristic) score of 98.9 %.

**Spectrogram-Based PD Diagnosis:** Zahid et al. (2020) [87] pre-sented a method based on spectrograms to diagnose PD. They used data from the Spanish PC-GITA dataset and proposed three approaches. The first approach involved transforming voice samples into spectrograms and utilizing a pre-trained CNN like ALEXNET to extract features. The second approach used the same pre-trained CNN model to extract speech features. The third approach incorporated information from spectral, statistical, and basic acoustic signals. Their MLP classifier achieved an impressive accuracy of 99.3 %.

**Dynamic Speech Features for PD Identification:** Quan et al. (2021) [83] studied static and dynamic speech features for PD identification. They collected data from a mixed-gender database comprising 15 healthy controls (HC) and 30 PD patients. The analysis revealed differences in the funda-mental frequency curve trajectories and articulation alterations between HC speakers and PD patients. They recommended the collection of time-series dynamic speech data for PD identification using a Bi-LSTM model. Dy- namic speech properties were quantified by calculating the energy content in voiced-to-unvoiced and voiced-to-voiced transitions. Experimental results showed that this approach improved PD recognition accuracy compared to existing ML models that rely on static features.

### 4.2 Review based on handwritings

**Deep Transfer Learning for Handwriting Datasets:** Deep *trans*-fer learning techniques performed exceptionally well in the HandPD and NewHandPD datasets, achieving an accuracy of 99.22 %. However, in the HandPD-MultiMC dataset, CNN with BLSTM outperformed with an accu-racy of 97.62 %. In idiopathic and PD datasets, DNN approaches demon-strated state-of-the-art results, achieving 99.1 % accuracy.

**PaHaw Handwriting Datasets:** For the PaHaw handwriting datasets, CNN achieved a maximum accuracy of 86.67 % when each feature's accuracy was evaluated and discriminated Parkinson's patients from healthy patients using a linear SVM classifier. On the other hand, the kNN algorithm had the lowest accuracy of 66.6 % [45].

**Combined Handwriting Databases:** Combining data from multi-ple handwriting databases, including PaHaW, HandPD, NewHandPD, and Parkinson's Drawing Dataset, improved the accuracy to 99.22 % [132]. This improvement was achieved by employing deep transfer learning-based algo-rithms with increased feature space and various data pre-processing tech-niques.

**Gait and Digital Device Dataset:** Using a dataset acquired from digital devices, a maximum accuracy of 99.37 % was achieved with DNN and gait dynamics [55]. The model was based on information from various sensors located under the feet of individuals. Additionally, a model based on Bi-GRU, including several pen-based handwriting characteristics, achieved a minimum accuracy of 79.64 %.

**Handwriting Features for PD Classification:** Handwriting samples from individuals with and without PD were analyzed for various features. These features were used as input for ML algorithms. SVM with a radial Gaussian kernel achieved an accuracy of 88.13 % in classifying PD based on handwriting features [34].

**Fig. 9:**
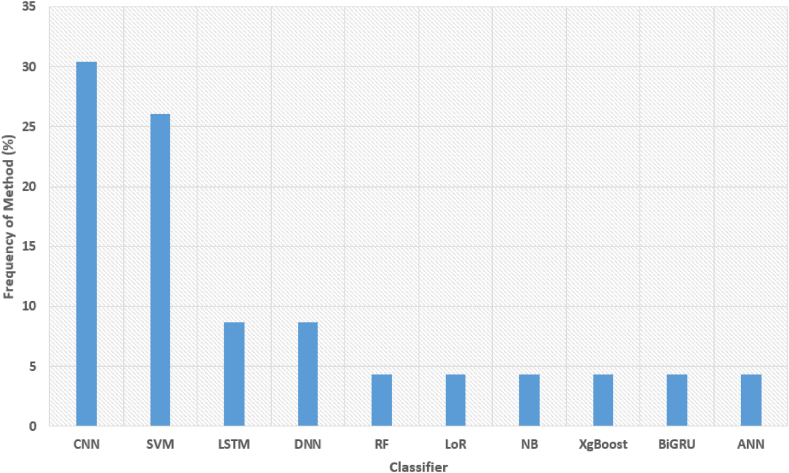
Number of methods (in %) used in handwriting dataset.

Fig. 9 shows the overall scenario where the CNN method has been used many times in various papers.

### 4.3 Control implementation for Parkinson's disease

**Low-Cost FPGA Implementation of Basal Ganglia Circuitry:** Yang et al. (2014) [120] focused on the activity of the basal ganglia-thalamocortical circuitry under Parkinsonian conditions. The study proposed a cost-efficient FPGA implementation of this circuitry, which could be used for real-time control of Parkinson's disease. The FPGA implementation was capable of replicating the dynamics of the basal ganglia-thalamocortical circuitry.

**Real-Time Control of Thalamocortical Circuitry:** In the study conducted by Yang et al. (2015) [8], the thalamocortical circuitry in PD was controlled in real-time using an FPGA-based platform. The research also proposed a digital implementation of thalamocortical neuron models. The FPGA implementation demonstrated the ability to reduce tremors and improve motor control in a simulated model.

**Real-Time Estimating System for Thalamocortical Parkinsonian Characteristics:** Yang et al. (2022) [137] suggested an efficient FPGA-based implementation of a real-time estimating system for the hidden Parkin-sonian features within the thalamocortical circuitry. The technology was designed to control PD symptoms in a closed-loop manner and was highly accurate at estimating the circuitry's status.

FPGA-based control methods hold promise for managing PD because they offer timely and cost-effective solutions for the complex neural circuitry involved in the disease. These implementations have the potential to provide real-time control and symptom management. However, further research is needed to validate the practical viability of these approaches and to enhance their effectiveness for addressing specific PD symptoms.

### 4.4 Practical implications of the paper

**Fig. 10:**
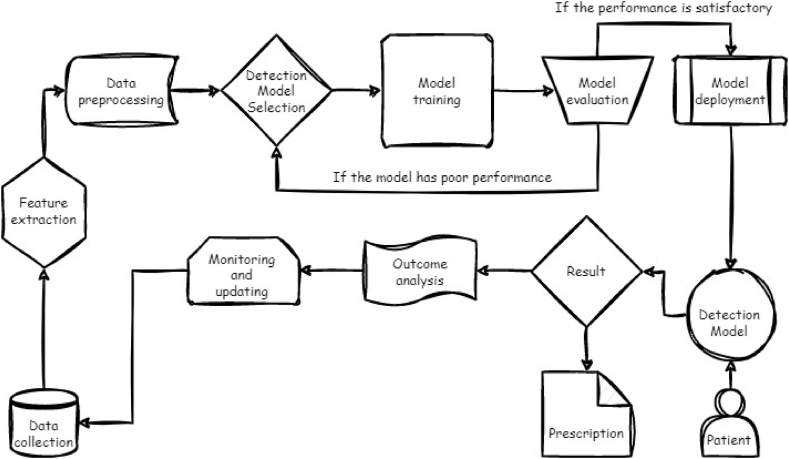
Designing practical implications of a machine learning-based Parkinson's dis-ease prediction system.

The ML-based system for predicting PD typically follows a multi-phase process, as depicted in the block diagram presented in Fig. 10. It begins with the collection of relevant data, encompassing patient demographics, medical records, and genetic information. Subsequently, feature extraction techniques are applied to distill critical attributes from the data, such as tremor frequency, gait characteristics, and speech quality. To streamline the process and reduce computational complexity, feature subset selection may be employed, particularly when dealing with vocal data. Data cleaning and preprocessing steps come next, involving tasks like handling missing values, feature selection, and data normalization to prepare the dataset for model-ing. The choice of a suitable ML or DL model, such as CNNs, LR, or DT, is a pivotal step in the process. Once the model is selected, it is trained using the preprocessed data. The performance of the trained model is then evaluated using standard metrics like accuracy, precision, recall, and F1 score. Subse-quently, the model is deployed to predict whether new patients may have PD. To ensure ongoing accuracy and generalizability, the model's performance is continuously monitored, and it is periodically updated with fresh data. Fi-nally, the results of the prediction system can be translated into practical clinical applications, such as early PD identification, remote patient mon-itoring, and telemedicine, potentially enhancing diagnosis, treatment, and patient outcomes. Additionally, various studies and practical applications underscore the transformative potential of machine learning and deep learn-ing in PD diagnosis and management. These applications range from RF- based diagnosis models to handwriting analysis tools, all contributing to early detection, accurate assessment, and improved quality of life for individuals with PD.

Several practical applications and studies have demonstrated the poten-tial of ML and data analysis in the context of PD. One such application, based on the work proposed by Xu et al. (2020) [129], involves the devel-opment of a computer-aided diagnosis model. This model utilizes sensor data from a smart pen and employs techniques like Principal Component Analysis (PCA) and RF classifiers to reduce data dimensions. Its practical implementation offers a promising tool for assisting clinicians in diagnosing PD, given its simplicity and reduced parameter requirements. By streamlin-ing the diagnosis process, it can help doctors make quicker and more precise assessments, ultimately leading to improved patient outcomes.

Moreover, studies conducted by Rosenblum et al. (2013) [23] and Li et al. (2017) [107] have explored the use of handwriting analysis as a reliable and cost-effective method to distinguish PD patients from healthy individuals. This non-invasive approach holds significant potential for early PD diagno-sis, particularly in high-risk groups. Analyzing an individual's handwriting can serve as a safe and accessible means of detecting PD at an early stage, po-tentially facilitating prompt intervention and treatment, thereby enhancing patient prognosis.

Furthermore, Drotar et al. (2016) [34] introduced an approach that assesses various kinematic aspects of handwriting, including stroke speed, length, trajectory, and jerk. These parameters can be leveraged to gauge the severity of PD and monitor its progression over time accurately. This method provides clinicians and researchers with a precise tool for evaluating the mo-tor symptoms of PD, aiding in both diagnosis and treatment. Additionally, the introduction of the levodopa equivalent dose (LED) measurement in the study allows for the comparison of different treatment options and personal-ized medication adjustments, enhancing the management of PD symptoms. Collectively, these studies contribute to the development of valuable tools and methodologies for assessing and managing PD motor symptoms. They hold the potential to improve patients' quality of life and inform the de-velopment of new treatment strategies. These innovations may lead to the creation of easy-to-use, non-invasive diagnostic tools for PD that can be read-ily integrated into clinical settings. Furthermore, they offer opportunities for screening individuals at risk of developing PD, enabling early detection and intervention. In conclusion, these advancements have significant implications for the identification, treatment, and management of Parkinson's disease.

### 4.5 Identification of research gaps

#### 4.5.1 Limitations of existing research on Parkinson diseases detection us-ing handwriting datasets

The analysis of handwritten movements as a means of PD detection of-fers a promising avenue for early diagnosis and monitoring. However, it is essential to acknowledge and address several limitations that have been iden-tified in previous studies, which can affect the accuracy and reliability of this approach.

One critical limitation is the small size of the datasets used in many studies. Limited dataset sizes, as noted in studies like [45,138], and [47], can lead to overfitting and may not fully capture the diversity of PD symptoms across a broader population. Addressing this limitation requires access to larger and more representative datasets to enhance the generalizability of PD detection models.

Another notable limitation is the reliance on a limited set of features for PD detection. Studies like [34,58] have highlighted the use of a restricted feature set, potentially missing out on crucial indicators of PD.

Expanding the feature set and exploring more comprehensive feature engi-neering techniques could contribute to more accurate PD detection.

ML algorithm selection is another area of concern. Some studies, such as [19], have used a high percentage of their dataset for training, which may lead to biased results. Moreover, relying on a single ML algorithm, as seen in Refs. [25,34], can limit the potential accuracy of the approach. Future research should consider more robust data splitting strategies and explore the performance of multiple ML algorithms to ensure comprehensive PD detection.

Dataset-specific limitations have also been identified, such as the absence of certain features like the Z coordinate in the PaHaW database, as men-tioned in Ref. [47]. Dataset-specific constraints can impact the transferability and applicability of models to real-world scenarios. Researchers should work on enhancing dataset completeness and relevance to the target population.

Additionally, various studies have focused exclusively on specific aspects of PD, such as tremor characteristics, gait data, or micrographia, as reported in Refs. [21,95,133], and [63]. While these approaches provide valuable insights, a more comprehensive approach that considers multiple symptoms and fea-tures may improve the overall accuracy of PD detection.

#### 4.5.2 Limitations of existing research on Parkinson diseases detection us-ing UCI dataset

One of the primary limitations is the small size of the datasets used in many studies, as highlighted in research conducted by Wu et al. (2017) [31] and Azad et al. (2013) [68]. Small datasets may not fully represent the diversity of PD symptoms and can lead to overfitting. Expanding the dataset size and diversity, perhaps through multi-center collaborations, would enhance the reliability of PD detection models.

Another limitation is the reliance on a limited number of features and al-gorithms. Some studies conducted by Lahmiri et al. (2019) [36] and Mostafa et al. (2020) [62], have used a restricted set of features and algorithms, potentially missing out on more robust diagnostic indicators and methods. Future research should explore a wider array of features and utilize a broader spectrum of ML and DL algorithms to improve accuracy.

Imbalanced datasets, as mentioned by Alissa et al. (2022) [82], pose a significant challenge in PD detection. Imbalanced datasets can lead to biased model predictions, as they favor the majority class. Techniques such as oversampling the minority class or employing specialized algorithms for imbalanced data should be considered to address this issue.

Variability in the lengths of time-series sequences within the dataset, as noted by Moharkan et al. (2017) [116], presents a practical challenge for DL models. Ensuring a consistent input size or exploring sequence-to-sequence models can help overcome this limitation.

The computational cost associated with deep learning algorithms, high-lighted by Senturk et al. (2020) [38], is indeed a concern. Finding ways to optimize training processes, such as using GPU acceleration or model com-pression techniques, can mitigate this challenge.

Lastly, enhancing dataset variability, as suggested by Naranjo et al. (2016) [40], is crucial. Ensuring that the dataset includes diverse voice recordings from different individuals and across various stages of PD will lead to more robust and generalizable models.

### 4.6 Critical analysis of ML and DL based techniques and the key gaps to Detect PD

ML techniques have been increasingly explored for PD detection in recent years. However, there are still gaps in the research, and this critical analysis aims to discuss these gaps in the context of ML techniques for PD detection.

#### 4.6.1 Lack of standardization in data collection and preprocessing

Many studies use different sources of data, such as clinical assessments, neuroimaging, and wearable devices, and the methods used for data prepro-cessing vary widely, leading to variability in results across different studies. For example, in a systematic review of ML and DL techniques for PD diagno-sis, Ashhar et al. (2021) [139] found that the studies included in their review used a wide range of data sources, including clinical scales, voice recordings, and accelerometer data, and the preprocessing steps varied significantly.

#### 4.6.2 Limited size and diversity of datasets

Most studies on PD detection using ML techniques have used small and homogeneous datasets, limiting the generalizability of their findings. For example, in a review of deep learning for healthcare, Lin et al. (2021) [140] noted that many studies on PD diagnosis using ML techniques have small sample sizes, and most of the data comes from a single center. This limited sample size can lead to overfitting and difficulty in generalizing the findings to other populations.

#### 4.6.3 Lack of exploration of PD subtypes

While many studies have focused on distinguishing between PD patients and healthy controls, few have explored the use of ML techniques for subtype classification. For example, in a review of subtypes of PD and their implica-tions for disease progression, Bluett et al. (2021) [141] noted that there are different subtypes of PD, and accurately classifying these subtypes is essen-tial for personalized treatment and management of the disease. However, few studies have explored the use of ML techniques for subtype classification.

#### 4.6.4 Lack of transparency in ML models

Many studies have used complex ML models, such as DL, that are difficult to interpret, limiting their clinical applicability. For example, Lin et al. (2021) [140] noted that deep learning models are often viewed as black boxes, and it can be challenging to understand how the models are making their predictions. This lack of transparency can make it difficult for clinicians to trust the results obtained from ML models and limit their use in clinical practice.

#### 4.6.5 Focus on supervised learning approaches

Most studies on PD detection using ML techniques have focused on su-pervised learning approaches that require labeled data for model training. However, labeling data can be time-consuming and expensive, and there is a need for unsupervised learning approaches that can learn from unlabeled data. For example, Ashhar et al. (2021) [139] noted that few studies have explored unsupervised learning approaches for PD diagnosis using ML tech-niques, highlighting the need for more research in this area.

### 4.7 Quality evaluation of included articles

Soumaya et al. (2019) [63] employed evolutionary algorithms, specifi-cally the GA and the SVM, renowned for their efficacy in decision-making processes. Leveraging the DWT, the speech signal underwent transformation, enabling the extraction of crucial features such as linear predictive coding (LPC), energy, zero-crossing rate (ZCR), Mel frequency cepstral coefficient (MFCC), and wavelet Shannon entropy. These features were derived from the approximation a3. Daubechies wavelets from the wavelet family were delib-erately chosen based on previous research, specifically opting for Daubechies level 2 at the third scale for superior outcomes. The study prioritized the low-frequency component signal, represented by the approximation a3, for pre-processing. The selection of tools, encompassing the genetic algorithm, SVM, and specific wavelet choices, was meticulously made, aligning with their suitability for the study's design and their effectiveness in extracting pertinent features from the speech signal.

The approach proposed by Tuncer et al. (2020) [52] holds pivotal signif-icance in PD diagnosis due to its potential for a more accessible examination compared to alternative methods. However, the paramount concern lies in ensuring accurate diagnoses among affected individuals. In the realm of computer-aided diagnosis, it is imperative to minimize or eliminate errors, specifically avoiding scenarios where (1) patients with PD are classified as healthy, and (2) healthy individuals are misclassified as having PD. The for-mer is particularly critical as delayed treatment may occur until a correct di-agnosis is established. The latter situation is also severe, signifying an incor-rect diagnosis. To mitigate these issues, selection criteria were meticulously adopted to identify the extractor and classifier combination that strikes a bal-ance between the aforementioned scenarios, thus reducing the occurrence of such cases. The evaluation of this balance was conducted through the anal-ysis of the confusion matrix. Addressing the common issue of unbalanced data, particularly the higher number of samples with the disease compared to healthy control samples, the study contends that this imbalance does not significantly impact the prediction model for the more prevalent class. The dataset was split, employing the hold-out technique with 75 % of samples used for training and 25 % for testing, as cross-validation was deemed impractical due to the dataset's substantial size.

Quan et al. (2021) [83] compares DL models using dynamic speech features with an end-to-end DL using a CNN model. The end-to-end DL outperformed DL models with dynamic features for sustained monophonic /a/input but showed a decrease in accuracy for a short sentence input. The study suggests potential enhancements, such as using a Multiscale CNN or incorporating processes like onset and offset transitions detection or speech signal rolling and filtering, to improve performance. The study addresses concerns related to biased results in performance evaluation using leave-one- out cross-validation by employing a dataset split into training and testing sets without sample overlap of one individual. Hyperparameter tuning is applied to explore the performance of DL models further, indicating sub-stantial improvement, especially with Bidirectional LSTM. Combining Bidi-rectional LSTM with dynamic speech features and end-to-end DL with CNN model results in a more robust PD detection system, enhancing flexibility across different input contents. While acknowledging the potential for more complex DL model architectures, the study suggests further exploration. It emphasizes the impact of speech features on classification performance, ad-vocating for the incorporation of additional features and their combinations to expect performance improvements. The study refrains from directly com-paring results with other studies due to differences in subjects, languages, input content, and preprocessing strategies. Instead, it focuses on objec-tively comparing the performance of different ML models within the same experimental environment.

Three FS methods were evaluated by Senturk et al. (2020) [38], demon-strating varying performance across different classifiers. The optimal com-bination of FS method and classification method was determined for PD diagnosis. The result illustrates the impact of FS methods on classifica-tion performance, revealing significant improvements, such as about 13 % for SVM, approximately 11 % for ANN, and around 5 % for Classification and Regression Trees (CART). It is emphasized that classification performance is also influenced by the parameters of classifiers. For SVM, parameters like c and gamma need appropriate determination, while for ANN, considerations include the number of hidden layers, neurons in hidden layers, activation functions, learning rate, momentum coefficient, normalization of data, epoch number, and more. The PD diagnosis system proposed in this study stands out due to its unique FS method, a streamlined feature extraction process, and the selection of a classifier. The system achieved high classification ac-curacy, showcasing the effectiveness of using voice features in PD diagnosis. Voice feature extraction is considered more accessible and cost-effective com-pared to MRI or motion-based methods. The study found that SVM with Recursive Feature Elimination (RFE) provided the best classification accu-racy, indicating that a specific subset of voice features contributes to more accurate PD patient classification. This approach reduces the computational cost associated with feature extraction and classification.

Pattern ranking techniques were employed as a preprocessing step by Lah-miri et al. (2019) [36] to evaluate the importance of patterns before induc-tion. The study considered both non-wrapper techniques (fast and simple) and one wrapper-based technique, SVM-REF-CBR, which employs recursive feature elimination (RFE). The computational cost of wrapper techniques, particularly SVM-REF-CBR, was higher than non-wrapper techniques. Re- sults showed that SVM achieved the highest classification accuracy (92.21 %) with the first fourteen voice patterns identified by the Wilcoxon-based pat-tern ranking technique. Different pattern ranking techniques led to variations in sensitivity, specificity, and accuracy. The ROC-based pattern ranking tech-nique yielded the highest sensitivity (99.63 %) with one voice pattern, high-est specificity (82.79 %) with thirteen voice patterns, and the second-best accuracy (92.13 %). The study emphasized that decreasing the number of phonation features led to small improvements in sensitivity and specificity. Comparisons with other studies suggested that systems for PD detection based on speech outperformed those based on MRI, emotions, and handwrit-ing characteristics. The study highlighted the need to explore multimodal feature-based systems for PD diagnosis, suggesting potential improvements in accuracy.

Oh et al. (2020) [81] introduces a web-based diagnosis technique for the future, involving the use of the Internet to diagnose PD patients. The EEG signals are collected, stored, and processed through a cloud-based CNN model, with the diagnosis sent back to the clinic, and potentially to the pa-tient via text message. The advantages and disadvantages of the proposed technique are outlined, with future plans to use a larger database and extend the application to detect other brain abnormalities. The discussion mentions the recognition rates achieved in earlier studies by the research group Pereira et al. (2016) [25] proposed handcrafted features from images extracted from the exams and obtained recognition rates of 65.88 % and 66.36 % for spirals and meanders, respectively. Later works by the same group [27] utilized fea-tures learned from CNNs on time-series images, achieving accuracies around 84.42 % and 83.77 % for meanders and spirals, respectively. Another study [25] fine-tuned a CNN using Bat Algorithm and achieved around 84.35 % recognition rates for meanders. The current study's approach, involving the combination of data from different exams using CNNs for feature learning, achieved an accuracy of nearly 93.50 %, surpassing the recognition rates of the previous works. The authors highlight the promising nature of mapping handwritten dynamics to time-series images for feature learning, especially when considering different exams for combination purposes. Baseline ap-proaches working on raw data and GLCM features did not yield satisfactory results, suggesting that mapping signals to images requires a more robust classifier capable of capturing both spatial and temporal information. CNNs, with their ability to capture information from different levels, are noted for providing high-dimensional feature vectors. Combining information from dif-ferent sources significantly reduces the difference among recognition rates for various techniques. This suggests that combining data from multiple sources can enhance the overall recognition performance and improve the accuracy of individual classifiers that may perform poorly on their own.

## 5 Future directions

**Fig. 11:**
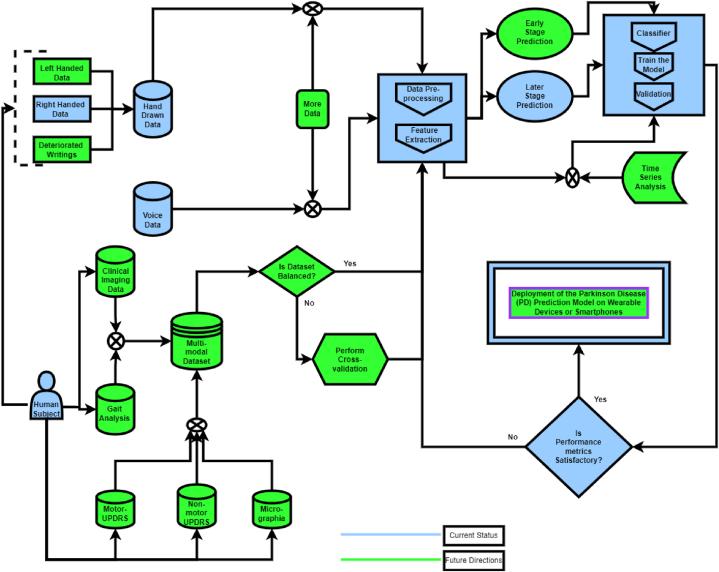
Future research directions of the parkinson's disease detection methods based on machine learning and deep learning algorithms.

The future research directions can contribute to the development of more accurate, personalized, and effective diagnostic tools for PD detection, which can improve the quality of life for patients and facilitate the development of new treatments. Fig. 11 shows the future research directions of the PD detection methods based on ML and DL algorithms.

### 5.1 Dataset improvement

#### 5.1.1 Dataset Reformation

The dataset used in the previous studies is relatively small, and the de-tection performance may vary on different voice datasets or with different types of handwriting samples. The performance and generalizability of the future model can be evaluated by putting it to the test on a larger dataset. A larger dataset could provide more robust results. Other sorts of data, such as imaging or clinical data, have not been taken into account in the previ-ous studies; the researchers primarily focus on utilizing sensor signals from a smart pen. Also, the results may not apply to left-handed people since the studies only included those who were right-handed.

#### 5.1.2 Balanced data

In contrast to other study sectors, getting real-world data from patients is recognised to be the toughest thing to achieve in the healthcare profes-sion. Any neurodegenerative condition's medical datasets are often imbal-anced. Cross-validation can also be used to address the issue of an unbalanced dataset. By eliminating traits that don't really help with PD detection, the feature sets can be tested and improved.

#### 5.1.3 Incorporation of multimodal data

Another promising direction is to incorporate multimodal data, such as voice, handwriting, gait, and eye movement data etc, to enhance the per-formance of the models. This is because DL techniques are getting better at merging with nature-inspired methods, which makes it hard to handle the very unbalanced dataset the studies have now because it affects the out-come. This approach can provide more comprehensive and diverse informa-tion about the patients' symptoms and motor functions, which can be used to develop more accurate and personalized diagnostic tools. For example, Muthuraman et al. (2021) [142] proposed a multimodal approach that com-bines voice, gait, and eye movement data to detect early-stage Parkinson's disease with high accuracy.

#### 5.1.4 Integration of longitudinal data

The PD is a progressive disorder that can exhibit a wide range of symp-toms and motor impairments over time. Therefore, it is important to in-corporate longitudinal data, such as repeated measurements of the patients’ symptoms and motor functions, to track the disease progression and pre-dict the future outcomes. This approach can also provide more insights into the underlying mechanisms of the disease and facilitate the development of personalized treatments. For example, Nilashi et al. (2016) [41] proposed a longitudinal DL model that integrates longitudinal clinical and neuroimaging data to predict the cognitive decline in PD patients.

### 5.2 Study area

#### 5.2.1 Gait analysis, speaker recognition, and emotion detection

The previous studies only focused on sustained phonation and speech tasks and hand-drawn spiral waves, which may not fully represent the com-plexity of patterns in PD. The study only evaluates the performance of ML methods on voice signals and hand drawn writings and spirals. It is suggested that a better choice would be to look into other possible signs of PD, such as gait analysis, speaker recognition, and emotion detection. Future research should also look into whether handwriting exercises can tell PD apart from other movement disorders and if they could be used as long-term biomarkers for the condition.

#### 5.2.2 Additional parameters and methods

Our next research projects will focus on a more comprehensive strategy in light of the changing field of PD diagnoses. While handwriting and voice datasets are our primary emphasis, we acknowledge the potential of adding other diagnostic factors, like gait analysis, facial expression identification, and tremor quantification. A deeper and more complex diagnostic framework is anticipated when these modalities function in concert. By comparing their performance to well-known diagnostic tools, we also hope to dive further into advanced ML and DL techniques. Our study will be at the vanguard of PD diagnostic innovation thanks to this two-pronged strategy, which includes improved analytical methodologies and increased data sources.

#### 5.2.3 Combining medical imaging or blood tests

The studies only focus on early-stage PD patients, and the model's per-formance may not be the same for patients in later stages of the disease. The previous methods have been tested on a limited number of voice dis- orders, and its performance on other voice disorders is unknown. It is also unknown if the handwriting tasks can tell PD from other illnesses because people with other movement disorders were not allowed to take part in the studies. The previous methods can be used in combination with other diag-nostic tools, such as medical imaging or blood tests, to improve the accuracy of the diagnosis.

#### 5.2.4 Exploring electromyography (EMG) or eye-tracking

The Total-Unified PD Rating Scale (UPDRS) and Motor-UPDRS predic-tions offer significant potential for assessing and monitoring motor symptoms in PD patients. To fully leverage this potential, ongoing research is explor-ing clustering, dimensionality reduction, and prediction approaches. These methods can extend beyond UPDRS assessments to tasks like drawing or tapping, broadening the evaluation of motor symptoms. Combining these techniques with other modalities such as electromyography (EMG) or eye-tracking can provide a more comprehensive understanding of PD's motor ef-fects. Additionally, these methods can be valuable for monitoring treatment effects, assessing motor function in other neurological conditions, integrating into wearable devices or apps for real-time health tracking, and supporting clinical trials to evaluate new PD therapies.

#### 5.2.5 Cognitive decline or depression analysis

The studies only take into account the motor symptoms of PD and ignore other non-motor symptoms, such as cognitive decline or depression, which can also have an impact on patients’ quality of life. To better understand the sensitivity, specificity, and underlying mechanisms, more investigation of the relationships between the cognitive and motor components of PD and handwriting metrics during sentence handwriting, both in the ON and OFF medication circumstances, is required.

#### 5.2.6 Disease severity on deterioration in handwriting

The study did not investigate the effect of disease severity on handwrit-ing measures, which could be an important factor in diagnosing PD. The previous methods are based on the analysis of handwriting, which may not be applicable to all PD patients, especially those who do not experience deterioration in handwriting.

#### 5.2.7 Measurement of micrographia

Investigating the use of ML based algorithms to classify different subtypes of micrographia, as the condition can manifest in different ways for different individuals. Exploring the use of wearable sensors to monitor handwriting movements in real-time, which could provide more objective and accurate measures of micrographia.

#### 5.2.8 Time series analysis

Future work will mix the original exam image with the time-series version. Future researchers may use auto-encoders after CNNs to reduce feature space dimensionality. Big data is omnipresent and can be utilized to analyze and predict the future. Most data about health care is unstructured and can be stored in a central location so it can be analyzed. Merging unstructured and structured data can improve medical care at a low cost. So, the future researchers may sort the data and look for patterns to predict future diseases so that doctors can catch them early.

#### 5.2.9 Experimental setup

The previous studies only use two types of microphones, which may not represent the full range of microphones used in clinical settings. The per-formance of the proposed approach could be evaluated on a wider range of microphones. The proposed method depends on the caliber of the input voice signals, and background noise or other factors that affect the caliber of the voice signals may have an impact on performance. The proposed model depends on how well the digitizing tablet technology used to capture the handwriting samples works, and any mistakes or inconsistencies in the data could change the results.

#### 5.2.10 Quality of signal

Also, the accuracy of the classification may depend on the quality of the signal sent in and the features that are extracted. The proposed method can be built into a mobile health system so that voice-based disease detection and monitoring can happen in real time. It is uncertain whether the handwriting tasks can be utilized as longitudinal biomarkers for PD because the study did not evaluate the consistency of the handwriting tasks across time.

#### 5.2.11 Effects of medication or other outside factors

The study doesn't look at how changes in medication or other outside factors might affect the kinematic aspects of handwriting, which could affect how accurate the measurements are. Even though it's important to use the right criteria to judge how well ML models classify PD, there is still room for improvement. For example, EEG signals have limited spatial resolution and are full of artifacts because of the way biomedical parameters are set up. For example, motion artifacts and the background noise might lower the quality of the voice in speech signals, leading to a wrong diagnosis of PD.

#### 5.2.12 Feature extraction

This paper suggests that one possible next step for this work is to look into how handwriting-based features could be combined with speech or gait analysis to improve the accuracy of PD diagnoses. The proposed method depends on how well the feature extraction process works, which can be affected by how good the images are that are sent in. Exploring the use of other feature extraction techniques such as the discrete cosine transform (DCT) and linear discriminant analysis (LDA) to improve the accuracy of the classification model.

### 5.3 Classification models

The study suggests doing more research on how to improve categoriza-tion performance by making hybrid deep learning models. Using sensor data from a smart pen, this could lead to an even better way to predict Parkin-son's disease. Investigating the use of other evolutionary algorithms such as particle swarm optimization (PSO) and ant colony optimization (ACO) in combination with SVM for speech signal classification.

In order to lower the dimensionality of the feature space, the report makes suggestions for further research, including integrating the original image from the exam with the time-series-based version and using auto-encoders imme-diately following CNNs. With these concepts, the suggested method for determining if someone has PD ought to become even more precise.

#### 5.3.1 Development of hybrid models

As mentioned earlier, the performance of existing models can be limited due to various factors, such as the size and quality of the dataset, the choice of features, and the type of algorithms used. Therefore, one possible future direction is to develop hybrid models that combine different types of fea-tures, algorithms, and data sources to improve the accuracy and robustness of the models. For instance Ref. [143], proposed a hybrid model that integrates clinical data, neuroimaging data, and deep learning algorithms to diagnose Parkinson's disease with high accuracy.

#### 5.3.2 Exploration of novel deep learning architectures

It is feasible that new architectures and approaches can be created to enhance the performance of PD detection models because DL has demon-strated considerable potential in a variety of applications, including medical image analysis and natural language processing. In order to extract features from brain MRI data for the diagnosis of PD [144], introduced a unique DL architecture dubbed GATA-Net that blends attention processes and graph convolutional networks (GCN).

#### 5.3.3 Transfer learning

A promising future direction for Parkinson's disease detection using *trans*-fer learning is to explore the effectiveness of different transfer learning meth-ods, such as domain adaptation, feature extraction, and model fine-tuning, in improving the performance of PD diagnosis models. Additionally, it is crucial to investigate the generalization capability of the transfer learning models, particularly when dealing with data from different sources or patient cohorts. Recent studies have demonstrated the potential of transfer learn-ing in improving the performance of PD diagnosis models. For instance, [107] proposed a deep transfer learning framework for PD diagnosis based on speech signals. They showed that pre-training the model on a large-scale speech recognition task significantly improved the accuracy of PD detection. Similarly [145], utilized transfer learning to improve the performance of a CNN-based model for PD diagnosis using PET images.

#### 5.3.4 Graph convolutional networks (GCN)

GCNs are a promising technique for analyzing complex data such as med-ical images and signals. Future research on the use of GCN in PD detection could focus on developing novel GCN architectures and integrating multi-modal data sources such as voice, gait, and neuroimaging data to improve the accuracy of PD diagnosis. Recent studies have demonstrated the poten-tial of GCN-based models for detecting PD using brain imaging data [146] and for differentiating PD from atypical parkinsonian syndromes using mul-timodal neuroimaging data [100]. Furthermore, transfer learning could be used to improve the performance of GCN models by pre-training on large, publicly available datasets such as the Human Connectome Project (HCP) or the Alzheimer's Disease Neuroimaging Initiative (ADNI) [69]. Overall, the use of GCN in PD detection holds great potential for improving the ac-curacy and efficiency of diagnosis, and future research should aim to explore and optimize its use.

## 6 Limitations

This article primarily focuses on the early identification of PD using ML and DL techniques. However, it does have certain limitations that need to be acknowledged. Firstly, the study's scope is limited as it only considers voice and hand-drawn spiral wave data, neglecting other potential sources of information like posture, facial expressions, or hand and eye movements. Consequently, this omission may introduce bias, especially in cases where individuals with PD exhibit symptoms in these unaccounted domains.

Furthermore, the study's scope is restricted to articles published in En-glish, potentially excluding valuable research conducted in other languages. Additionally, the review does not encompass large-scale, multi-centric studies that delve into subtyping or evaluating the severity of PD. These areas are crucial for a comprehensive understanding of the disease and may warrant future investigations.

Moreover, while this work offers a thorough assessment of existing re-search, it does not propose novel approaches or instruments for PD detection or assessment. Instead, it consolidates and evaluates previously conducted studies. It is important to note that there is no one-size-fits-all, clinically validated standard approach for PD detection or assessment. Thus, further research is needed to develop more reliable and accurate tools in this domain. Collecting data from real-life PD patients is a challenging task due to the nature of neurodegenerative disorders. Additionally, imbalanced medical datasets are common in this field, potentially skewing the results of machine learning models.

## 7 Conclusion

This review work has shed light on the significant potential of ML and DL techniques in advancing the field of PD diagnosis. Early and accurate diagnosis of PD is essential for timely intervention and tailored treatment, and ML or DL approaches offer promising avenues to achieve these objectives. Through an extensive analysis of voice, handwriting, and wave spiral datasets, we have demonstrated that ML and DL algorithms have the ca-pacity to significantly enhance diagnostic accuracy. Various classifiers and models have been explored, showcasing their effectiveness in distinguishing.

PD patients from healthy individuals. Moreover, the potential identification of specific biomarkers through these techniques holds promise for further im-proving diagnostic precision and understanding the underlying mechanisms of PD.

Our review has encompassed a range of data formats and commonly uti-lized ML or DL methods in the context of PD diagnosis. While significant progress has been made, challenges remain, including standardizing data col-lection and preprocessing, addressing the imbalance in datasets, and ensuring transparency in ML models.

As the field continues to evolve, future research should focus on larger and more diverse datasets, the integration of multimodal data sources, and the incorporation of longitudinal data to track disease progression. Additionally, research efforts should extend to subtype classification, severity evaluation, and prognosis prediction of PD.

Ultimately, the integration of ML and DL-based tools into clinical prac-tice has the potential to revolutionize PD diagnosis, enhance patient care, and facilitate the development of personalized treatments. While further re-search and validation are needed, this review serves as a valuable roadmap for researchers and medical professionals striving to harness the power of ML and DL in the early identification and management of PD.

## CRediT authorship contribution statement

**Md.Ariful Islam:** Writing – original draft, Resources, Methodology. **Md.Ziaul Hasan Majumder:** Writing – original draft, Visualization, Resources, Investigation, Formal analysis. **Md.Alomgeer Hussein:** Writing – review & editing, Supervision, Resources, Investigation. **Khondoker Murad Hossain:** Writing – review & editing, Visualization, Investigation. **Md.Sohel Miah:** Formal analysis, Data curation, Conceptualization.

## Declaration of competing interest

The authors declare that they have no known competing financial interests or personal relationships that could have appeared to influence the work reported in this paper.
